# Guidelines for DNA recombination and repair studies: Mechanistic assays of DNA repair processes

**DOI:** 10.15698/mic2019.01.665

**Published:** 2019-01-07

**Authors:** Hannah L Klein, Kenny K.H. Ang, Michelle R. Arkin, Emily C. Beckwitt, Yi-Hsuan Chang, Jun Fan, Youngho Kwon, Michael J. Morten, Sucheta Mukherjee, Oliver J. Pambos, Hafez el Sayyed, Elizabeth S. Thrall, João P. Vieira-da-Rocha, Quan Wang, Shuang Wang, Hsin-Yi Yeh, Julie S. Biteen, Peter Chi, Wolf-Dietrich Heyer, Achillefs N. Kapanidis, Joseph J. Loparo, Terence R. Strick, Patrick Sung, Bennett Van Houten, Hengyao Niu, Eli Rothenberg

**Affiliations:** 1 New York University School of Medicine, Department of Biochemistry and Molecular Pharmacology, New York, NY 10016, USA.; 2 Small Molecule Discovery Center and Department of Pharmaceutical Chemistry, University of California, San Francisco, California 94143, USA.; 3 Program in Molecular Biophysics and Structural Biology, University of Pittsburgh, Pittsburgh, PA 15261, USA.; 4 The University of Pittsburgh Cancer Institute, Hillman Cancer Center, Pittsburgh, PA 15213, USA.; 5 Institute of Biochemical Sciences, National Taiwan University, NO. 1, Section 4, Roosevelt Road, Taipei 10617, Taiwan.; 6 Biological Physics Research Group, Clarendon Laboratory, Department of Physics, University of Oxford, Oxford, OX1 3PU, UK.; 7 Department of Molecular Biophysics and Biochemistry, Yale University School of Medicine, New Haven, CT 06520, USA.; 8 Department of Biochemistry and Structural Biology, University of Texas Health San Antonio, San Antonio, Texas 78229, USA.; 9 Department of Microbiology and Molecular Genetics, University of California, Davis, CA 95616, USA.; 10 Department of Biological Chemistry and Molecular Pharmacology, Harvard Medical School, 250 Longwood Avenue, Boston, MA 02115, USA.; 11 Department of Molecular and Cellular Biochemistry, Indiana University, Bloomington, IN 47405, USA.; 12 Ecole Normale Supérieure, Institut de Biologie de l'Ecole Normale Supérieure (IBENS), CNRS, INSERM, PSL Research University, 75005 Paris, France.; 13 Institut Jacques Monod, CNRS, UMR7592, University Paris Diderot, Sorbonne Paris Cité F-75205 Paris, France.; 14 Departments of Chemistry and Biophysics, University of Michigan, Ann Arbor, MI 48109, USA.; 15 Institute of Biological Chemistry, Academia Sinica, 128 Academia Road, Section 2, Nankang, Taipei 11529, Taiwan.; 16 Department of Molecular and Cellular Biology, University of California, Davis, CA 95616, USA.; 17 Programme Equipe Labellisées, Ligue Contre le Cancer, 75013 Paris, France.; 18 Program in Molecular Biophysics and Structural Biology, University of Pittsburgh, Pittsburgh, PA 15261, USA.; 19 Department of Pharmacology and Chemical Biology, University of Pittsburgh, Pittsburgh, PA 15261, USA.

**Keywords:** chromatin dynamics, chromosome rearrangements, crossovers, DNA breaks, DNA helicases, DNA repair centers, DNA repair synthesis, DNA resection, double strand break repair, DSBs, endonuclease protection assay, genome instability, gross chromosome rearrangements, fluorescent proteins, FRET, homologous recombination, mismatch repair, nonhomologous end joining, nucleotide excision repair, PALM, photoactivated fluorescent proteins, recombinase filament assembly, single-molecule, single-particle tracking, super resolution, structure-selective endonucleases, synthesis-dependent strand annealing, transcription coupled repair

## Abstract

Genomes are constantly in flux, undergoing changes due to recombination, repair and mutagenesis. *In vivo*, many of such changes are studies using reporters for specific types of changes, or through cytological studies that detect changes at the single-cell level. Single molecule assays, which are reviewed here, can detect transient intermediates and dynamics of events. Biochemical assays allow detailed investigation of the DNA and protein activities of each step in a repair, recombination or mutagenesis event. Each type of assay is a powerful tool but each comes with its particular advantages and limitations. Here the most commonly used assays are reviewed, discussed, and presented as the guidelines for future studies.

## INTRODUCTION

Genomes are constantly subject to DNA damage arising from endogenous and exogenous sources that result in single or double stranded breaks, modified bases, and chromatin changes, among others. To protect the genome, cells have an arsenal of repair mechanisms to sue, the specific mechanism dependent on the type of damage and its context. Our understanding of the myriad repair pathways has come from genetic studies to identify genes encoding proteins for DNA repair and the consequences of loss of these functions, *in vivo* genetic and physical assays to determine the consequences of failure to repair, cytological assays to interrogate protein interactions and real time events, and *in vitro* biochemical assays to determine the substrate and repair events, and the molecular intermediates in repair.

In a separate guideline article, we have reviewed genetic, molecular and cytological assays for repair. In this guideline article mechanistic assays are presented, specifically single molecule assays and biochemical assays. Single molecule assays can be applied to *in vivo* or *in vitro* situations. Single molecule fluorescence and PALM (photoactivated localization microscopy) imaging are used to study the position and dynamics of tagged proteins interacting with DNA substrates that are induced by external stimuli. Movement of proteins on DNA molecules, using DNA tightropes or DNA nanomanipulation and a magnetic trap allows visualization of DNA topology changes resulting from protein interaction with the DNA molecules. Both types of approaches have led to a detailed understanding of repair processes and in some cases have challenged the current models of repair.

Biochemical assays permit detailed investigation of DNA protein interactions. Reactions mimicking the proposed intermediates in homologous recombination (HR) are the focus of the guidelines here. From the initial step in recombination, the assembly of the presynaptic filament to the formation of the D-loop, followed by extension of the D-loop from the primer terminus, these reactions are studied *in vitro* using substrates and purified proteins. The proposed intermediates are often derived from *in vivo* genetic experiments and tested *in vitro*. The *in vitro* results then inform further *in vivo* biological experiments. HR involves DNA helicases and nucleases. Assays for helicases are included here, which represented key steps in the HR process. Finally, structure-selective endonucleases are needed at several steps in the HR process. Here, different types of substrates and assays for joint molecule resolution are presented.

These guidelines should be useful for the application of these approaches to many areas of DNA repair. Individual author contributions and contact information are available in Supplementary Table 1.

## SINGLE MOLECULE ASSAYS FOCUSING ON DNA REPAIR

Single molecule assays are powerful tools that can be used to investigate the activity of proteins on DNA. They bypass the need to synchronize initiation events and enable the detection of transient intermediates that are otherwise lost to ensemble averaging. This section describes several single molecule techniques and some of the insights into DNA repair that have been directly made from the minute level of detail that these assays are able to provide (**Box1**).

BOX 1: **SINGLE MOLECULE ASSAYS FOCUSING ON DNA REPAIR**

**DNA tightropes to watch repair proteins interrogate DNA |** The method of DNA tightropes to directly visualize proteins interacting with DNA substrates is described. Advantages of this method are presented with examples of target searches by DNA repair proteins**.**

**Single-molecule (Förster resonance energy transfer) FRET illuminates the non-homologous end joining process in vitro |** smFRET is used to study the details of NHEJ and deduce causes of aberrant end joining.

**Single molecule imaging to study mismatch repair in living cells |** Live cell single-molecule fluorescence is used to study MutS in bacterial cells. The positioning and dynamics of proteins can be assessed and responses to external stimuli determined to understand a repair process at the nanometer scale.

**Single molecule DNA nanomanipulation |** Use of a magnetic trap to observe real-time changes in DNA topology and structure from protein interactions. Here it is used to study MutS in bacteria.

**Single molecule PALM imaging |** A description of PALM and its application to translesion polymerases in living bacterial cells is presented.

**Tracking-PALM direct single-molecule imaging |** Combining single-molecule tracking with PALM has led to a localizationbased super-resolution imaging method. Here use of this method to study DNA repair in living bacteria is presented.

### Dancing on DNA tightropes: watching repair proteins interrogate DNA in real time

**Figure 1:**
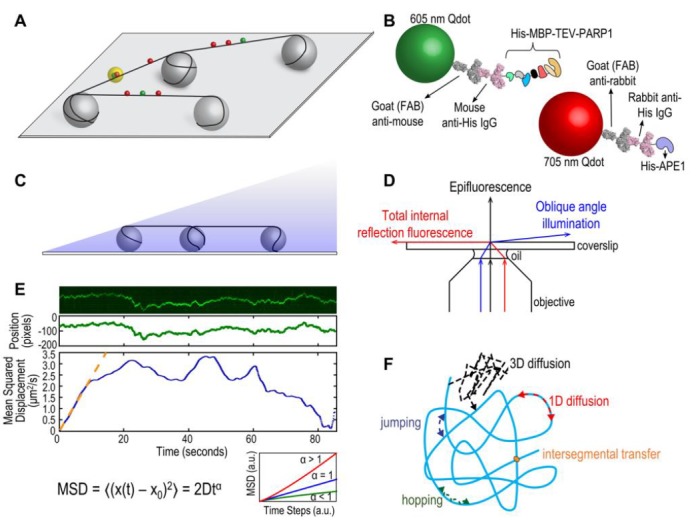
FIGURE 1: (A) Schematic of DNA tightrope setup. Long DNA molecules are suspended between 5 μm poly-L-lysine-coated silica beads on a glass coverslip. Qdot-labeled proteins bound to DNA shown in red and green (see B); colocalized particles highlighted in yellow. Adapted with permission from [13]. **(B) Two orthogonal Qdot-protein labeling strategies.** Top: A 605 nm Qdot (green) with conjugated anti-mouse secondary antibody (grey) bound to a mouse anti-His primary antibody (pink), bound to a His-tagged protein. Bottom: A 705 nm Qdot (red) with conjugated anti-rabbit secondary antibody (grey) bound to a rabbit anti-His primary antibody (pink), bound to a His-tagged protein. Adapted with permission from [13]. **(C) DNA tightropes in a flow cell with oblique angle illumination.** Adapted with permission from [8]. **(D) Ray diagram showing incident laser light paths for epifluorescence (black), TIRF at the critical angle (red), and oblique angle illumination (blue).** Adapted with permission from [8]. **(E) Top:** Sample kymograph of a Qdot-labeled protein displaying random linear diffusion on a DNA tightrope. Y axis, position; X axis, time. **Middle:** 1D Gaussian fittings of the light intensity profile at each time point of the above kymograph shown as position in pixels vs. time. 1 pixel = 46 nm. **Bottom:** Mean squared displacement (MSD) vs. time. The initial linear portion of the MSD plot is fit to the equation MSD = 2Dt^α^ (orange line). Inset: Sub-types of 1D diffusion defined by α values. Super-diffusion (red), random diffusion (blue), sub-diffusion (green). Adapted with permission from [11]. **(F) Modes of protein-DNA interaction.** Search strategies typically involve some combination of: 3D diffusion in solution (black), 1D linear diffusion (red), jumping (blue) or hopping (green) between DNA segments, and intersegmental transfer (orange). Adapted with permission from [11].

In order to understand how DNA repair proteins find damaged sites in a vast excess of non-damaged DNA, the field of DNA repair has moved to various single molecule approaches allowing direct visualization of proteins interacting with their DNA substrates [1]. These single molecule techniques can provide unique insights into population trends without losing detailed information on individual particles or events [2]. An optical platform consisting of DNA tightropes was developed by Neil Kad at the University of Vermont and first used to study bacterial nucleotide excision repair (NER) proteins [3, 4] and base excision repair (BER) glycosylases [5]. This DNA tightrope assay takes a similar approach to the DNA curtain setup developed by Dr. Eric Greene and colleagues [6, 7] with one important difference. The tightrope itself is established by suspending long molecules of double stranded (ds) DNA (∼90% contour length) between poly-L-lysine coated five micron beads dispersed in a flow cell (**Figure 1A**). Visualizing repair proteins of interest up off the surface requires labels with bright fluorescent signals, and real-time imaging requires photostability over long periods. To accomplish these two needs, repair proteins are conjugated to quantum dots (Qdots) with appropriate antibodies (**Figure 1B**) and added to the flow cell. Interactions are recorded in real time, in the absence of flow, using oblique angle fluorescence on a total internal reflection fluorescence (TIRF) microscope with a CMOS or EMCCD camera (**Figure 1C, D**) [8]. Here, we will discuss the advantages and limitations of the DNA tightrope assay, current applications, and potential new directions.

The tightrope assay has its own exceptional strengths. Bringing the DNA up off the bottom of the flow cell overcomes surface interactions that can arise from DNA being in contact with a phospholipid layer, as well as it assures the observer that the Qdots being monitored are attached to DNA repair proteins engaged with the DNA and not proteins or Qdots simply sticking to the surface. Because the DNA is suspended on both ends, once the proteins of interest are added, they can be observed in the absence of flow. Finally, this optical platform allows for the use of long DNA substrates and the potential to engineer multiple site-specific lesions that can be marked with Qdots [8]. The use of Qdots, however, also presents some potential challenges.

Relatively bulky labeling strategies using large Qdots and antibodies (**Figure 1B**) may sterically hinder protein interactions with DNA and/or other proteins. Despite this potential problem, we have been able to observe three-color Qdot-labeled NER UvrABC complexes moving together on DNA [9]. Controls of non-conjugated Qdots and optimization of protein:antibody:Qdot ratios are required for such experiments. The size of the Qdot (∼10-15 nm) and inherent rotational drag must also be considered when analyzing the diffusive behaviors of proteins on DNA. The use of oblique angle illumination enhances signal-to-noise over epifluorescence microscopy and resolution can be further improved by fitting Gaussians to the intensity profiles along a kymograph (**Figure 1E**). Movies can be collected as fast as 100 frames per second and the mean positional accuracy for a Qdot-labeled protein has been reported as 6 ± 3 nm [10].

The DNA tightrope assay can be used to answer several major questions about protein-DNA interactions. First, and perhaps most importantly for this method, is the question of modes of target search (**Figure 1F**) [11]. Resolution limits prevent observation of very short-range motion below 100-200 bp, but motion above this scale can be investigated in depth. Movies of protein-DNA interactions are converted to kymographs and subsequent mathematical analyses of observed linear diffusive behavior can provide insight into the molecular basis for these interactions. Mean square displacement analysis of particle motion is used to calculate the diffusion coefficient *D* and anomalous diffusion exponent α, providing information about rates and nature of the diffusion process (**Figure 1E**). Surprisingly we have found that several repair proteins, including Rad4 [12] and PARP1 [13], undergo anomalous diffusion, showing highly constrained motion around the site of damage. In addition, Dr. Susan Wallace's group has shown that aromatic side chains of BER glycosylases caused pausing at damaged sites in DNA [5, 14]. Furthermore, the cohesion protein SA1 was observed to alternate between fast and slow diffusion and this was dependent on telomeric sequences used in the DNA tightropes [15].

The use of orthogonal labeling strategies (i.e. Qdots with distinct emission spectra and conjugation schemes, **Figure 1B**) can be used to answer questions about colocalization and other interactions on DNA. Dimerization or interaction of two (or more) DNA repair proteins can be observed by separate and different labeling of the proteins of interest. Furthermore, such experiments can detect changes in dynamic behavior of proteins in the absence/presence of other DNA repair partners. For example, the eukaryotic NER recognition protein UV-DDB was observed to dimerize on UV-damaged DNA and abasic DNA [10]. In another example, UvrB was only observed binding to DNA tightropes in complexes containing UvrC or UvrA [9, 16]. To determine if proteins colocalize with target lesions, site specific arrays of DNA damage can be engineered with a biotinylated base proximal to the lesion and labeled with a streptavidin-coated Qdot orthologous from the labeled proteins [8]. In this way, UV-DDB [10] and PARP1 [13] were observed colocalizing with abasic sites along DNA tightropes. However, limits in spatial resolution dictate that direct interactions should be confirmed with complementary methods.

The DNA tightrope assay has made important contributions to the study of DNA repair proteins from both microbial systems and more complex multicellular organisms. Use of this optical platform will continue to foster progress in the field as the method is improved and modified to suit newer needs. For example, assembly of nucleosomes along DNA can be used to study chromatin [17]. Furthermore, incubation of DNA tightropes with nuclear extracts will allow for the study of specifically labeled proteins in the context of all their interacting partners [18]. The future holds great promise as single molecule detection of DNA repair proteins dancing on DNA occurs in even more physiologically relevant settings, and even within living *Escherichia coli* cells [19].

### Single-molecule (Förster resonance energy transfer) FRET illuminates the non-homologous end joining process *in vitro*

#### Overview

The central premise behind single-molecule experiments is to avoid losing information through ensemble averaging. DNA:protein interactions are well suited to be studied at a single-molecule resolution, in part, due to the relatively facile isolation and detection of individual DNA molecules. Chromosomal double strand breaks (DSBs) are arguably the most cytotoxic form of DNA damage, and are fatal to a cell if left unchecked. Non-homologous end joining (NHEJ) dominates over HR during G1 in mammalian cells, most notably due to the lack of a sister chromatid template to complete HR, but it is known to generate errors that are also extremely damaging to the cell [20, 21]. DSBs can produce DNA ends with varied chemistries, and the NHEJ machinery includes end processing enzymes to efficiently join different types of broken ends together [22–24]. However, there are certain DNA substrates that are more prone to incorrectly repair DSBs, and the reasons behind this are still unclear. Single-molecule Förster resonance energy transfer (smFRET) experiments using total internal reflection fluorescence microscopy (TIRFm) are ideally equipped to accurately quantify rate constants and identify transient intermediates that are otherwise hidden in an ensemble. smFRET is therefore well suited to illuminate the subtleties of the NHEJ mechanism and deduce the causes of aberrant end joining.

#### Description of method/assay

To study NHEJ using smFRET, fluorescently labeled DNA substrates can be immobilized to a surface, and the intensity of the fluorescent dyes can be recorded throughout the end joining process [21]. The two pieces of DNA are labeled with two different fluorophores, Cy3 and Cy5, which act as an energy donor and acceptor respectively. FRET is only likely to occur when these dyes are close to each other, therefore the FRET response can be interpreted in terms of the relative distance between two linear DNA molecules, which are analogous to the ends produced by a DSB.

A sample chamber is created between a coverslip and glass slide, and the internal walls are passivated by a PEG surface to minimize non-specific binding. DNA is covalently bound to a biotin moiety which interacts with neutravidin molecules on the modified surface of the glass coverslip [25]. Single-molecule resolution is achieved by only sparsely populating the slide with an immobilized DNA substrate so that each pixel corresponds to a region on the slide with only one fluorophore [26]. Typically, an incubation of low picomolar concentrations of the biotinylated DNA is sufficient to produce a surface that is populated by many, but distinct, DNA molecules. The number of immobilized DNA molecules in each pixel on screen can be confirmed by photobleaching experiments to show that the majority of high intensity spots measure the emission from a single dye only. A second DNA structure can then be introduced to the sample chamber, along with the necessary proteins to carry out the end joining process.

**Figure 2:**
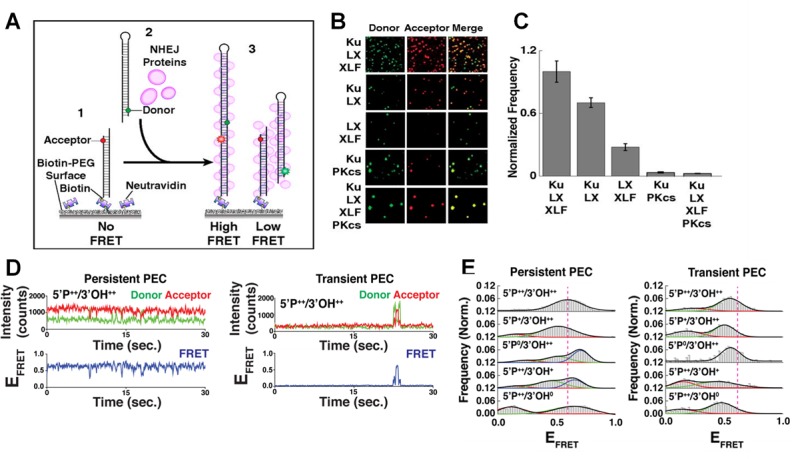
FIGURE 2: smFRET of NHEJ synapsis and ligation. **(A)** Schematic of the end joining smFRET assay. (1) dsDNA labelled with an acceptor dye is immobilizes to a PEG surface. (2) NHEJ proteins and a second dsDNA molecule, labelled with a donor, are added to the solution. (3) As the two ends of the DNA are joined together, FRET is observed. **(B)** Images showing the donor/acceptor channels for experiments that are investigating different combinations of NHEJ proteins. **(C)** The quantification of the number of spots in end joining experiments, from n>1000 molecules for each condition. Error bars illustrate SEM. **(D)** Typical smFRET traces for persistent and transient PECs. **(E)** Frequency distributions for FRET values of persistent and transient PECs observed with various end chemistries of dsDNAs used in the end joining assays. Reproduced from Ried *et al.* (2015 and 2017) [21, 27].

The initial joining of two DNA ends by NHEJ proteins form a paired end complex (PEC) as shown in **Figure 2A**, and can be monitored in a number of ways: the number of FRET pairs observed can be used to quantify the yield of the end joining reaction; the changes in FRET efficiency during PEC formation allows the movement of the DNA ends to be observed; and the measurement of the dwell times in between these movements can infer the stability of the PEC [21, 27]. Single-molecule microscopy is able to capture these small but significant structural changes in real time, since cameras, such as electron multiplying charge coupled devices (EMCCD), can resolve events that take place over a few milliseconds [26]. This temporal resolution is complemented by changes in FRET efficiencies able to identify structural movements within the sub-nanometer regime. In order to increase the signal:noise ratio, the fluorescent signal is amplified over the background fluorescence by using an evanescent field to predominately excite the fluorophores nearest the PEG surface [28]. This field is produced by total internal reflection, and effectively limits the volume of the sample chamber that is illuminated by the laser, therefore reducing the background intensities of other fluorophores not specifically bound to the surface. There is a wide choice of donor and acceptor dyes that are commercially available for single-molecule experiments. In addition to Cy3 and Cy5, there are alternative dyes such as the Atto- or Alexa- series that also display highly stable photophysics required under constant illumination. Buffer additives, such as oxygen scavenging systems and anti-blinking reagents, prolong the lifetime of fluorescent dyes and ensure stable emission of the fluorophores [29, 30]. The anti-correlation between donor and acceptor intensities during changes in FRET facilitates more accurate recognition of structural movement compared to simple thresholding algorithms, since any decrease in the donor intensity must coincide with an increase in the intensity of the acceptor. Without a concomitant change in both fluorophores, changes in intensities are attributed to experimental noise and do not affect the dwell time analysis [31].

Many individual end joining events are analyzed to build frequency distributions and gain reliable statistics concerning the dynamics and organization of NHEJ [27]. It was found that efficient NHEJ was most reliant on a core complex that involved the Ku70/80 heterodimer (Ku), XRCC4, XLF and DNA Ligase IV (L4) [21, 32]. The yield of the NHEJ reaction was not improved by the addition of DNA-dependent protein kinase catalytic subunit (DNA-PKcs) as shown in **Figure 2B-C**, which in turn promoted the gathering of multiple paired donor complexes, in agreement with DNA-PKcs' role in the repair of clustered DSBs [21].

The initial formation of the PEC in the presence of Ku, XRCC4, XLF and L4 displayed fluctuations between different FRET states as the protein machinery sampled various configurations to align the two ends correctly [21]. This was observed both in the broad distribution of FRET states and the instability of individual FRET trajectories. The sampling was also confirmed when the position of the dyes were changed to produce a high FRET state when the DNA molecules were arranged adjacent to each other, and not stacked linearly on top of one another [21]. Again, these FRET trajectories also fluctuated between different states, as the NHEJ machinery searched to correctly position the DNA ends for ligation.

The action of the NHEJ core complex on a ‘simple' DSB was compared to DNA ends which had either the 5′ phosphate (P) or 3′ hydroxyl (OH) groups removed [27]. There was a significantly larger reduction in the yield of NHEJ after the removal of the 5′P compared to the removal of the 3′OH. This removal did not affect protein binding to the DNA; therefore the decrease in NHEJ was attributed to a less efficient end pairing of two DNA strands. Further NHEJ assays with L4 mutants, designed to inhibit ligation, also severely reduced pairing confirming that this was the rate limiting step during NHEJ [27].

The PECs can be grouped into two classes depending on their stability [27]. Transient PECs demonstrated rapid dissociation, and typically sampled conformations that produced lower FRET states compared to the persistent PECs (**Figure 2D-E**). For both transient and persistent complexes, attempts to perform NHEJ on DNA ends lacking 5′P or 3′OH resulted in shifted FRET values compared to the simple DSB, therefore disrupting the end joining process [27]. If such errors occur *in vivo*, the dissociation of the transient PEC would allow accessory NHEJ factors to modify the DNA, and NHEJ could be completed upon the next attempt by the core complex.

The iterative nature of NHEJ was exaggerated when the overhang regions of the DNA substrates were mismatched, resulting in the core complex struggling to adopt a stable configuration [22]. This behavior was reversed and returned to favor high FRET intermediates when an insert of L4, that is responsible for encircling the dsDNA at a strand break, was deleted [22]. The deletion accommodated the mismatch but at lower NHEJ pairing efficiencies. When NHEJ was attempted using complementary sequences embedded within mismatched overhangs, a wide distribution of FRET efficiencies from transient PECs was observed, from attempts to hybridize mismatched sequences before the PEC dissociated. Persistent PECs demonstrated higher FRET efficiencies than the transient PECs, suggesting that successful searches for complementary sequences produced DNA structures with single stranded (ss)DNA flaps, which are most likely removed *in vivo* by nucleases to complete NHEJ [22].

smFRET microscopy is therefore a powerful tool that can define the organization and kinetics of NHEJ proteins *in vitro*. It has been used to identify the core proteins required for efficient NHEJ and explored how the NHEJ complexes mediate pairing of dsDNA ends. This has led to insights into how NHEJ is a dynamic and iterative process, and also how it attempts to avoid joining incompatible ends through PECs that are sensitive to destabilization by mismatches and mutations to the core complex of NHEJ machinery.

#### Cautionary notes

The use of smFRET with immobilized substrates can be limited by a number of factors. In order to track the emission intensity over a prolonged period of time, in this instance, the substrates are required to be immobilized to a PEG surface. The proximity to the surface may have adverse effects on the activity of proteins and other biological molecules of interest. When completing experiments such as measuring the reaction yield of NHEJ by recording the density of fluorescent DNA on a slide, suitable controls must also be taken to guarantee that there is minimal non-specific binding to the surface [33].

Although the evanescent wave limits emission from fluorophores that are not adjacent to the surface, the concentration of highly fluorescent molecules in solution should be kept in low nanomolar regime to avoid lowering the signal:noise ratio. The immobilized fluorophores themselves are under constant bombardment of photons to be detected as single molecules, and each time the fluorophore absorbs a photon it carries the risk of temporarily or permanently entering a dark state. Oxygen scavenging systems and anti-blinking reagents are added to the imaging buffers but the experiment is typically limited to a few minutes before fluorophores are photobleached [30, 34, 35]. The rate of NHEJ and many other DNA/protein interactions are fast enough for smTIRFm to be used; however it is difficult to measure the rates of processes that take place over several minutes. It is also challenging to accurately resolve processes with very short dwell times (typically >10 ms) due to the limitations of the EMCCD [36]. It is therefore possible to identify when the NHEJ machinery searches for the correct alignment of DNA ends, but it is not possible to specifically define the transition states within that search.

The design of the experiment should also take into account the position of the fluorophores [26]. Labeling efficiencies are dependent on the availability of residues that are ideally exposed on the surface of target molecule, and the inter-dye distance (*r*) determines the FRET efficiencies that will be observed. Changes in FRET can only be detected typically between 1 and 9 nm; however due to the *r*^*-6*^ dependence of the energy transfer, FRET is most sensitive to changes for a smaller window of length. The ideal placement of dyes would ensure any changes in their position would center around the inter-dye distance that gives a FRET efficiency of 0.5 (*R*_*0*_), which is where FRET's dependence is most sensitive to *r*. Fluorescent dyes are also prone to interact with each other, and interactions such as dye-stacking can cause misleading changes in emission intensity so it is also preferable to avoid very short inter-dye distances [37]. The residue to be conjugated to the dye must be chosen carefully so that any changes will not disrupt the structure or activity of the protein or DNA structure of interest. Overall, care should be taken when choosing where to position the dyes to ensure efficient labeling and appropriate expected FRET values, whilst preventing other perturbations that will affect the active molecules.

#### Conclusion

The repertoire of single-molecule experiments includes a wide range of setups, including measuring force and studying individual molecules *in vivo*. Analyzing the fluorescence intensity of DNA molecules immobilized *in vitro* has a relatively long history, but remains a powerful tool to define the activity and structure of DNA and proteins. There are still large gaps in our knowledge concerning processes such as NHEJ and, despite its limitations, smFRET is able to provide highly detailed information that is vital to our understanding of how genomic integrity is maintained or mismanaged.

### Single-molecule imaging measures dynamics and localization to uncover the mechanism of DNA mismatch repair in living cells

#### Overview

The DNA mismatch repair (MMR) process corrects DNA base-pair mismatches that evade proofreading [38, 39]. During this highly conserved process, DNA replication errors must be found, identified, and corrected. In bacteria, MutS is the first protein involved in the MMR pathway, and it is responsible for detecting rare base-pairing errors; in eukaryotes this function is carried out by MutS homolog (MSH) proteins [40]. Recently, the extensive *in vitro* knowledge of the MMR process has been complemented by live-cell single-molecule fluorescence investigations of the MutS protein in *Bacillus subtilis* [41]. Overall, single-molecule imaging provides new insight into this mechanism because it can investigate the heterogeneities that complicate traditional ensemble approaches.

#### Description of method/assay

Live-cell single-molecule imaging tracks, localizes, and characterizes the fluorescence of fluorescent protein fusions inside cells to measure the positioning and dynamics of proteins inside cells [42]. Furthermore, based on protein mutations and responses to external stimuli, the mechanisms underlying these subcellular behaviors can be determined. Here, we describe some key techniques that were used to understand the nanometer-scale dynamical process that lie at the heart of MMR in *B. subtilis*.

#### Localization and co-localization in MMR

**Single-particle tracking/photo-activated localization microscopy (SPT-PALM).** MMR and DNA replication are intimately coupled in cells, and this coordination was understood with two-color (SPT-PALM) [43]. Single-molecule imaging was enabled by tagging with the photoactivatable fluorescent protein PAmCherry [44], which is initially dark until photoactivated by a 405-nm laser. *B. subtilis* strains natively expressing MutS fused to the photoactivatable fluorescent protein PAmCherry as the sole source of MutS were examined in an inverted microscope (Olympus IX71) coupled to an EMCCD camera (Photometrics Evolve) via appropriate filters. We exposed the cells to a 200-ms 405-nm pulse (Coherent 405-100); a power density of 120 W/cm^2^ was chosen such that 0 – 1 molecules per cell were photoactivated by this pulse and then imaged the photoactivated MutS-PAmCherry molecules with a 561-nm laser (Coherent Sapphire 561-50). This MutS-PAmCherry fusion was localized and tracked until photobleaching and then the cycle of 405-nm photoactivation and 561-nm imaging was repeated. To provide context for our observations of PAmCherry positioning and motion, we expressed MutS-PAmCherry in cells expressing fusions of the yellow fluorescent protein mCitrine to the β-clamp loader protein DnaX. This DnaX-mCitrine fusion was imaged under 488-nm laser illumination (Coherent Sapphire 488-50) to provide the location of the DNA replication machinery in each cell. The centroid position of the DnaX-mCitrine clusters, *r*, was measured *N* times, and the radius of gyration, 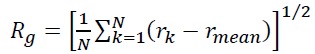 of the centroid position was calculated to be *R*_*g*_ = 84 nm, indicating that the *B. subtilis* replisomes are strongly confined [41].

**Figure 3:**
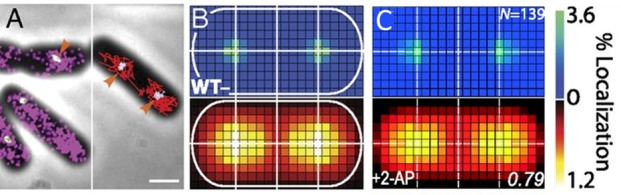
FIGURE 3: MutS localizations and co-localizations in *B. subtilis* cells. **(A)** Photoactivated localization microscopy (PALM) reconstruction (left; magenta) and single-molecule trajectories (right; red) of MutS-PAmCherry overlaid with DnaX-mCitrine (green and blue) and phase-contrast cell images. Overlapping signals are in white and orange arrows indicate replisome regions at which MutS enrichment is observed. (Scale bar: 1 μm). **(B)** Localization probability density maps of DnaX-mCitrine (upper; blue-green) and MutS-PAmCherry (lower; red-yellow) within a normalized cell. **(C)** Normalized cell maps as in (B) but for the MutS[F30A] mutant shows that the localization of MutS to the replisome is independent of the ability of MutS to identify mismatches. Reproduced from Liao et al. PNAS 2015 [41].

**Single-cell super-resolution images and localization probability density maps.** In each imaging frame, the MutS-PAmCherry peak is identified and localized based on a fit to a 2D symmetric Gaussian function. Each super-localized MutS-PAmCherry position can be mapped (**Figure 3A**; left), and sequential MutS-PAmCherry localizations are grouped into a trajectory (**Figure 3A**; right). Localization probability density maps were constructed from at least 100 single-cell experiments (**Figure 3B**). First, each cell was rotated such that its principal axes were aligned with the image frame, and coordinates of single-molecule localizations were normalized with respect to the rotated cell contour. Based on the probability of finding a molecule in a certain region within the cell, two 2D localization probability density maps were constructed for each cell, one for DnaX-mCitrine and the other for MutS-PAmCherry. The final density maps were obtained by averaging localization probabilities over all cells. Because the DNA replication and mismatch repair processes are symmetric in the *B. subtilis* cell, the maps were symmetrized with respect to the cell center [45]. These localization probability density maps show that MutS accumulates at the replisome even in the absence of significant DNA mismatch errors.

**Protein mutations and response to external perturbations.** The mechanism by which MutS identifies and responds to DNA mismatch errors was identified based on protein mutations and external perturbations. In particular, we measured the relationship between DNA replication and the position and dynamics of single MutS molecules based on four *B. subtilis* strains, each designed to impair one of four MMR steps: (i) MutS binding to β-clamp, (ii) mismatch recognition, (iii) MutS nucleotide binding, and (iv) subsequent MutL recruitment. Furthermore, the action of MutS was measured before and after treatment with the mismatch-forming drug 2-aminopurine (2-AP). Examining differences between these experimental conditions identified the mechanism of MutS localization to the replisome. For example, the localization pattern of MutS[F30A], which is unable to recognize mismatches, was compared to that of wild type (WT) MutS (**Figure 3C**). Both with and without 2-AP, this mutant preserved the elevated MutS density around the replisome observed in WT cells. Beyond these qualitative similarities, the comparison of colocalization between different cases was quantified by calculating the Pearson correlation coefficient between each pair of DnaX and MutS density maps. Here, upon 2-AP treatment, cells expressing WT MutS and MutS[F30A], show correlation coefficients of 0.81 and 0.79, respectively, indicating no measurable difference in localization. This colocalization measurement indicates that in *B. subtilis*, MutS localization to the replisome precedes—and occurs independently of—mismatch recognition.

#### The dynamics of MMR

**Figure 4:**
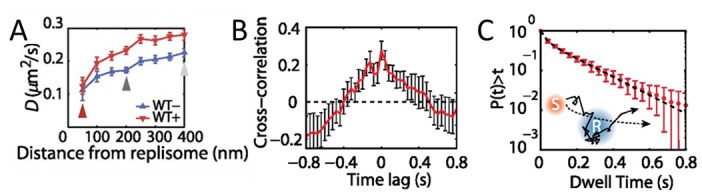
FIGURE 4: MutS dynamics in *B. subtilis* cells. **(A)** Effective diffusion coefficients, *D*, of MutS-PAmCherry as a function of separation distance from the nearest replisome. Error bars indicate 95% confidence interval. WT+ indicates cells that have been treated with the 2-AP mutagen. **(B)** Cross-correlation between the separation between MutS and the center of DnaX cluster and the instantaneous speed of MutS. **(C)** Cumulative probability distribution of the time, *t*, that MutS (red “S”) spends within the same replisome region (blue “R”). Reproduced from Liao et al. PNAS 2015 [41].

**MutS diffusion as a function of subcellular position.** Single-molecule tracking in living cells can measure dwell times to determine the binding kinetics of proteins in cells and changes with protein mutations and external perturbations can indicate mechanism. Though the probability density maps in **Figure 3B** indicate that on average, most of the MutS molecules accumulate at the replisome, the single-molecule trajectories in **Figure 3A** show that the MutS molecules diffuse throughout the entire cell before and after each dwell event. Furthermore, we observe that MutS diffuses rapidly far away from the replisome whereas, on entering the replisome region (separation distance < 100 nm), MutS slows down to match the average speed of DnaX. From the MutS trajectories, we calculated the average effective diffusion coefficient, *D*, of MutS as a function of separation distance from the nearest replisome (**Figure 4A**). These *D* values were calculated from the mean square displacement for over 3,000 trajectories longer than 10 frames. Both before and after treatment with the 2-AP mutagen, *D* decreases as the separation distance decreases. However, we found that MutS exhibits an overall faster motion after 2-AP treatment, consistent with *in vitro* observations that MutS switches from rotation-coupled sliding to a faster rotation-free sliding after mismatch binding [46]. These MutS dynamics can be further quantified by calculating the normalized cross-correlation coefficient [47] between MutS-DnaX separation and speed (**Figure 4B**).

**Dwell times and response to perturbations.** The dwelling times of MutS at the replisome indicate the time of interaction. To quantify how much time a MutS protein spends within the replisome region, we fit the cumulative probability, *P(t)>t*, of the dwell time, *t*, of MutS in the replisome region with a two-term exponential decay function, *P*(*t*) = *A*_1_ exp(−*t*/τ_1_) + *A*_2_ exp(−*t*/τ_2_) (**Figure 4C**) and obtained two dwell time constants of τ_1_ = 25 ms (*A*_1_ = 42%) and τ_2_ = 188 ms (*A*_2_ = 58%). This measurement indicates that, in the absence of induced mismatches, a WT MutS spends 188 ms at the replisome before it is “recycled” and replaced by another molecule. The fast 25 ms time constant represents MutS molecules that diffuse past the replisome without binding (dashed arrow in **Figure 4C**).

#### Cautionary notes

**The Effect of Labeling.** Though genetically encodable fluorescent proteins have revolutionized our understanding of cell biology, these tags are large (PAmCherry has a molecular weight of 28.8 kDa) and can perturb function. The activity of all labeled proteins must therefore be ascertained. Since MutS can respond to rifampin challenges in cells [48], the mutation rates of *B. subtilis* cells expressing either MutS, MutS-PAmCherry, or ΔMutS were compared after plating on and growth in 100 μg/mL rifampin. The low mutation rate of cells expressing MutS (100% MMR activity) was preserved for cells expressing MutS-PAmCherry (97% MMR activity), whereas the mutation rate increased 50-fold in ΔMutS (0% MMR activity). Thus we conclude that MutS-PAmCherry retains MMR activity.

**Dwell Times and Probe Photobleaching.** To calculate dwell time constants in **Figure 4C** required us to analyze only single MutS trajectories that started outside the replisome, remained trackable within the replisome, and ended outside the replisome. As a result, only MutS trajectories that start and end outside the replisome were measured, and the 188-ms dwell time constant represents a lower bound. Furthermore, though this measurement was well suited to characterize these relatively short MutS dwell times, the approach must be modified for extension to longer dwell times because of the limited photostability of fluorescent proteins. For instance, under continuous illumination, PAmCherry molecules can only be tracked for ∼750 ms before PAmCherry undergoes irreversible photobleaching. Thus, the imaging process must be modified to measure longer dwell times. For instance, to extend this method to measuring the dwell times of the DNA polymerase PolC-PAmCherry at the replisome [49], we therefore performed time-lapse imaging. In this time-lapse imaging mode, every frame is still captured with a 50-ms image integration time (τ_int_), but a time delay (τ_delay_) of 0 – 1.45 s is introduced between each pair of consecutive frames. The time-lapse period (τ_TL_ = τ_int_ + τ_delay_) extends the observable dwell times and enabled quantification of the much slower PAmCherry exchange dynamics: a dwell time of 0.97 s was measured.

#### Conclusion

Single-molecule methods have revealed new insight into the nanometer-scale dynamical nature of DNA mismatch repair in living cells. In particular, by examining the localization and motion of MutS in *B. subtilis,* we have understood how this mismatch repair protein efficiently identifies DNA mismatches. These experiments show that MutS must initiate mismatch binding close to the replisome and that mismatch detection increases MutS speed, likely due to sliding clamp formation after mismatch recognition.

### Single-molecule DNA nanomanipulation

**Figure 5:**
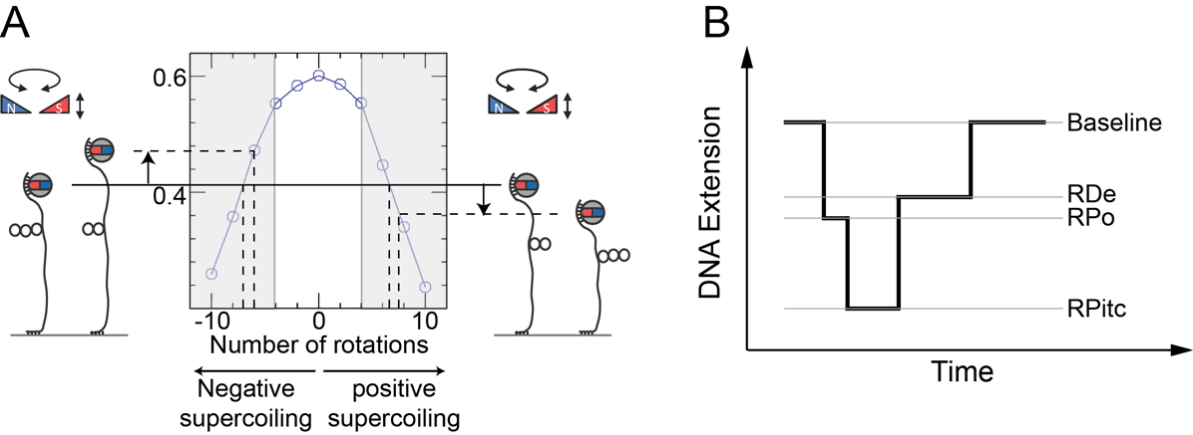
FIGURE 5: Single molecule DNA nanomanipulation. **(A)** Extension vs. supercoiling curve for a single tethered DNA molecule. A schematic diagram of the magnetic trap is provided, showing a pair of trapping magnets located above a coverslip-bound DNA molecule tethered at one end to a magnetic bead. **(B)** Real-time detection of transcription. A succession of three states (RNAP-promoter open complex, RPo; RNAP-promoter initially-transcribing complex, RPitc; and RNAP-DNA elongation complex, RDe) can be identified corresponding to the different stages of transcription. RPo is oftentimes too short-lived to be observed in the presence of high concentrations of nucleotides, as RNAP can begin transcribing in those conditions. The initial baseline state, in which no RNAP is actively unwinding the DNA, is recovered upon transcription termination once the RDe complex reaches the transcription termination sequence and is ejected from the DNA.

Essentially all protein-DNA interactions result in mechanical deformation of the DNA double helix. Single-molecule nanomanipulation based on the magnetic trap [50] is a method that allows one to observe in real-time the mechanical and topological changes imposed on DNA by interacting proteins [51–53], providing unique quantitative and mechanistic insights into the nature of their interaction. In this approach a dsDNA is tethered via multiple attachment points at one end to a magnetic bead and at the other end to a treated glass surface. The DNA-tethered bead is then placed under a magnetic trap (see **Figure 5**) allowing for controlled rotation of the bead as well as application of an extending force. The DNA is thus topologically constrained by the trap's magnetic field and by the multiple attachment points to bead and surface. The response of DNA to supercoiling and extending as imposed via the trap is reflected in real-time in the end-to-end extension of the DNA polymer, which can be determined by measuring the position of the tethered magnetic bead above the surface using videomicroscopy. Once the mechanical properties of DNA are calibrated via external means such as the magnetic trap, one can interpret protein-induced changes in DNA's mechanical properties to monitor protein-DNA interactions in real-time.

Mechanical calibration of DNA is relatively straightforward. First, at low applied force (F∼0.3 pN) the DNA is extended by ∼70% of its contour length. Then at this constant extending force the DNA responds to rotation of the trap's magnetic field by supercoiling and forming interwound looped structures named “plectonemes,” causing its extension to contract as depicted in **Figure 5A**. For both positive and negative supercoiling there exists a linear regime in which the DNA extension change is proportional to the topological change – on the order of 60 nm per turn, and with a force-dependence which scales as F^-0.4^ [54, 55]. Once the DNA's mechanical response to supercoiling and extending has been calibrated in this manner and found to be consistent with prior results, one typically imposes a fixed set of mechanical constraints (i.e. externally-imposed force and supercoiling) and then lets the enzyme of interest further modify the DNA's mechanical properties. These modifications will be reflected by changes in the position of the bead above the surface. One thus monitors in real-time the supercoiled DNA's extension changes as imposed by the interacting protein and from there, using the calibration data, works back to recover the topological changes imposed on the DNA molecule. For instance, unwinding of one turn of DNA (10.5 bp) results in topological annihilation of one plectonemic supercoil if the DNA substrate is initially negatively supercoiled, but the topological addition of a plectonemic supercoil if the DNA substrate is initially positively supercoiled. In the former case the DNA responds to unwinding by increasing its extension by ∼60 nm; in the latter case the DNA will reduce its extension by ∼60 nm. Detection of such signals allows one to begin to understand the underlying DNA deformation.

This approach has been fruitfully applied to the study of bacterial transcription as well as related processes such as transcription-coupled repair (TCR). Indeed, RNA polymerase (RNAP) imposes distinct topological (unwinding) states on DNA via formation of the so-called “transcription bubble” within which it templates nascent RNA production against the sequence of bases present on the so-called “non-coding” DNA strand. Transcription by RNAP has been characterized on both positively and negatively supercoiled DNA with the magnetic trap assay (**Figure 5B**) [52, 56]. To do so one must simply engineer into the DNA the relevant sequences for RNAP activity, namely: a promoter sequence, a transcript sequence, and an intrinsic terminator sequence. Upon engaging a single RNAP via the promoter sequence, the nanomanipulated DNA displays a series of distinct extension states, corresponding to a series of topological states, which reflect the different stages of transcription.

Transcription initiation begins with formation of the RNAP-promoter open complex (RPo) in which ∼12 bp of promoter DNA are unwound by the polymerase, causing a ∼70 nm change in DNA extension from the baseline state (**Figure 5B** and [52]). Next, initial synthesis of RNA takes place prior to bonafide promoter escape (i.e. dissociation of RNAP from the promoter). The RNAP-promoter initially-transcribing complex (RPitc) transiently unwinds additional downstream DNA and reels it into its active site to maintain register between nascent RNA and the template strand. This process, termed “scrunching,” results in transient unwinding of an additional ∼12 bp of DNA, for a total of ∼24 bp. As a consequence, there is a net change in DNA extension of ∼140 nm relative to the baseline state [56]. This state is followed by promoter escape and formation of an RNAP-DNA elongation complex (RDe) characterized by a ∼50 nm change in extension from the baseline state, corresponding to stable unwinding of ∼9 bp. Finally, upon completing productive transcription and reaching the transcription termination sequence, RNAP and RNA are released from DNA and the DNA extension returns to its baseline value [56].

For each individual transcription “pulse” generated by a single RNAP, each transcriptional sub-state described above is characterized by two numbers: the extent of the DNA deformation in that state, and the lifetime of the state. Different states display different lifetime distributions, depending on the number of rate-limiting steps that separate one state from the next. A state separated from the next by a single rate-limiting step displays a lifetime (or dwell-time) distribution which is single-exponential. A state separated from the next by a succession of multiple irreversible rate-limiting steps typically displays a Gaussian distribution of lifetimes. Thus, the lifetime of RPitc is found to obey single-exponential statistics, while the lifetime of RDe typically follows a Gaussian distribution reflecting polymerization of many bases between initiation and termination [52]. Representation of lifetimes and conformational states in a 2D plot provides a way to understand the correlation between the structural nature of the protein-DNA interaction and its kinetics. A variety of molecular transcription intermediates, caused by RNAP or by the action of additional proteins, has been detected and characterized through this method. This has allowed analysis of RNAP backtracking during promoter escape and its rescue by the GreA transcription factor [57]. It has also allowed for the detailed molecular characterization of bacterial transcription-coupled repair, a process wherein RNAP, stalled atop a DNA lesion on the transcribed strand, is remodeled and displaced from DNA by the Mfd translocase which thereafter recruits downstream repair factors UvrA and UvrB to the exposed lesion [58, 59].

**Figure 6:**
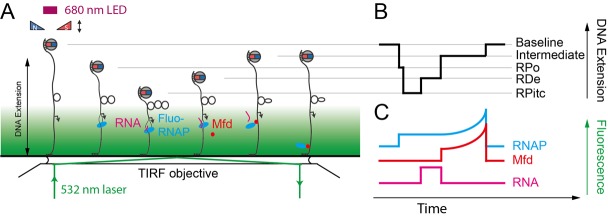
FIGURE 6: Sketch of the NanoCOSM assay (for nanomanipulation and colocalization of single-molecules) as applied to TCR. **(A)** An exponentially-decaying total-internal reflection field is added to the magnetic trap by introducing a laser line into the system (green line) such that it impinges, collimated, upon the glass-water interface of the sample at or above the critical angle σ_c_=sin^−1^(n_water_/n_glass_) − here σ_c_ is the beam angle relative to axis normal to the interface, and n_water_ and n_glass_ are the indices of refraction of water and glass, respectively. **(B)** Magnetic trapping of the DNA allows one to monitor transcription-coupled repair as remodeling by Mfd of RNAP stalled on a DNA lesion and the concomitant formation of an intermediate extension state (labeled ‘intermediate'). The molecular composition of the mechanically-defined intermediate state naturally remains ambiguous based on the magnetic trapping data; it could include Mfd, or RNAP, or both. **(C)** Simultaneous single-molecule fluorescence detection of fluorescently labeled components of the reaction lifts the ambiguity and allows one to enumerate the components present on the DNA at each stage of the repair reaction. These experiments show that Mfd remodels RNAP, causing it to lose hold of the nascent RNA, but that thereafter the RNAP stays associated with Mfd. Fluorescence signatures of RNAP and Mfd increase after remodeling because Mfd remains on the DNA and translocates in the same direction as initial transcription, i.e. towards the surface, transporting its associated RNAP along as this permits the translocase to remain tightly bound to the DNA and highly processive.

The single-molecule nanomanipulation assay is powerful for characterizing interactions between individual proteins and DNA, but this approach falls short if dynamic multi-protein complexes, of the kind that are often involved in DNA repair, are to be considered in depth. Indeed, the DNA conformation is a single metric which can potentially be impacted by each protein present in the reaction. Hence further determination of the composition of the active complex will provide even more details for interpreting the mechanisms underlying these deformation processes. Thus, single-molecule nanomanipulation is fruitfully combined with single-molecule fluorescence via total internal reflection microscopy methods (TIR) so that one can image fluorescently-labeled components while simultaneously manipulating DNA in the magnetic trap (**Figure 6**) [59, 60]. The combination of these two techniques correlates the DNA-state signal with the simultaneously-determined composition of the molecular reaction intermediate. Such correlative experiments carried out using i) fluorescently-labeled RNAP which had been stalled in elongation, ii) fluorescently-labeled Mfd, or iii) fluorescently labeled RNA, showed that Mfd displaces stalled RNAP from DNA, causing it to lose hold of the nascent RNA, but that RNAP thereafter remains attached to Mfd and acts as a processivity factor to maintain the Mfd repair protein on the DNA long enough for downstream components to have time to be recruited to Mfd (**Figure 5E**, [59, 60]).

Although these real-time nanomechanical assays provide extensive new information on molecular processes, there are limitations to their resolution. Ultimately the spatiotemporal resolution of such assays depends on parameters such as the size of the magnetic bead (typically 1 µm in diameter), the viscosity of water (which cannot be varied to any useful effect), and the stiffness of the DNA – itself a function of its length, extending force, and supercoiling. In the above experiments analyzing simple transcription the DNA is typically 2 kbp in length. The mean extension of such a DNA supercoiled by four turns and subjected to a 0.3 pN (pico-Newtons) force is on the order of ∼300 nm and it displays Gaussian fluctuations with a mean fluctuation (standard deviation) σ ∼ 40 nm and a characteristic timescale for the fluctuations, τ, on the 0.1 second timescale. Averaging the bead's position signal for T seconds thus allows one to collect n = T/τ independent measurements of the bead's position, giving a standard error on mean bead position of roughly σ/n^1/2^. For T = 1s the error on mean bead position is therefore ∼15 nm [61]. Because topological unwinding of ∼10 bp changes DNA topology by one unit of linking number (Lk) and therefore gives rise to a ∼60 nm change in DNA extension, this method allows one to study the interactions between supercoiled DNA and proteins of interest with a topological resolution of ∼2-3 bp on the second time-scale. DNA bending deformations – distinct from DNA torsional deformations – can further be monitored too within a few nanometers by comparing the conformational changes imposed by the protein on positively or negatively supercoiled DNA [52]. As a result of the low stiffness of the supercoiled DNA, the time resolution of this method is limited in the example given here to ∼1-2 seconds if one wishes to have resolution of a few bps' worth of unwinding for any given individual event. Of course, collecting many such events allows one to further reduce error via averaging, but this takes time and it is important for environmental conditions to remain constant throughout the measurement. We note that thermal regulation of the experimental system is particularly important given the strong temperature-dependence of DNA unwinding processes in general, and transcription in particular.

What can be done to improve the spatiotemporal resolution of the assay? Possibilities include reducing the size of the bead; stiffening the DNA by reducing its length; or stiffening the DNA by increasing the applied force. A force range of 10 fN (femto-Newtons) to 50 pN can typically be applied to 1 μm magnetic beads using the magnetic trap, but for transcription studies on supercoiled DNA a force on the order of 0.3 pN is typically used. Indeed, determination of the bead position above the surface is the central readout of this method, and lowering the force increases both the amplitude of Brownian fluctuations experienced by the bead as well as the fluctuations' characteristic timescale. This degrades spatiotemporal resolution as more extensive time-averaging is required to characterize the mean bead position to a predetermined resolution – the opposite of what we wish to achieve. On the other hand, if the force is increased significantly beyond 0.3 pN then negatively supercoiled DNA will begin to denature, rendering the substrate chemically inhomogeneous and allowing one to only perform experiments on positively supercoiled DNA. The force is thus already somewhat optimized for these specific assays (although it may be higher for others, depending on the application). Alternatively, one can increase the DNA stiffness by using a shorter DNA; some success has been obtained working with DNA molecules that are ∼1 kbp in length, but these experiments are challenging as the magnetic bead often ends up getting stuck on the surface to which it is so close. Furthermore, for combined nanomanipulation and fluorescence assays it is important to keep the bead from coming too close to the surface, and this typically requires working with DNA molecules 3 kbp in length. Finally, one can reduce the size of the bead to allow its Brownian fluctuations to have faster dynamics and thus be more rapidly averaged out. This, however, comes at the cost of lowering the applicable force range, as the maximum applied force depends on the size of the magnetic bead (and in particular the amount of paramagnetic ferrite it encapsulates).

The ability to supercoil DNA in a simple fashion using the magnetic trap offers one last important feature to consider for the study of DNA repair processes. Indeed, DNA repair processes such as NER or MMR involve at least one single-strand incision of the damaged DNA strand as a precursor to elimination of the damaged DNA base and resynthesis of fresh, intact DNA. Incision of DNA is readily observed in the supercoiling assay because it results in an essentially instantaneous loss of supercoils and a sudden, readily detectable, increase in DNA extension to the maximal extension state obtained in the absence of supercoils. This allows for kinetic characterization of the DNA repair events all the way through to DNA incision itself. Religating the incised DNA with an enzyme such as T4 DNA ligase regenerates the original dsDNA with the damage, permitting a new cycle of initial repair steps to take place. Single-molecule DNA nanomanipulation thus offers numerous avenues of study in the analysis of DNA repair processes, and the combination of magnetic trapping and single-molecule fluorescence appears as a useful new tool in the kit.

### Single-molecule PALM imaging of translesion polymerases in live bacterial cells

#### Overview

Translesion synthesis (TLS) is a DNA damage tolerance pathway that allows cells to bypass unrepaired DNA lesions that might otherwise block replication. In this process, specialized TLS polymerases are recruited to carry out synthesis past DNA lesions. Once the lesion is bypassed, the template is returned to a replicative polymerase so that normal DNA replication can continue. Although biochemical and genetic assays have provided great insight into the mechanisms of TLS in the model bacterium *E. coli*, they are unable to probe directly where and when TLS occurs in cells. Fluorescence imaging in live cells, by contrast, is well suited to reveal the localization and recruitment of TLS polymerases and to answer questions about when they are recruited to replication forks, whether they are recruited to other cellular sites, and what molecular interactions are required for recruitment. Here we describe the use of particle-tracking PALM in live *E. coli* cells, with an emphasis on using this method to study the cellular localization and recruitment of the TLS polymerase Pol IV [62].

#### Description of method/assay

PALM is a super-resolution imaging technique that can be combined with particle tracking to resolve the localization and dynamics of single TLS polymerases and other proteins in live bacterial cells [19, 62–64]. PALM takes advantages of specialized photoactivatable fluorescent proteins (PAFPs) that form initially in a dark state, in which they do not fluoresce under visible excitation. Proteins in a dark state can be converted to a bright state, or photoactivated, by near- UV excitation. Once converted to the bright state, proteins can be visualized with visible excitation until they irreversibly photobleach. Under excitation conditions in which no more than one protein is activated at a time, the motion of this molecule in the cell can be unambiguously tracked.

*E. coli* strains bearing protein fusions to PAFPs can be constructed using several approaches. In general, an endogenous chromosomal knock-in is preferable to ectopic or plasmid-based expression, especially when the protein copy number is expected to affect its function or cellular localization. Generally, a linker of approximately 5–20 amino acids is inserted between the PAFP and the protein of interest. λ Red recombineering [65] is a powerful tool for introducing these fusions into an *E. coli* strain background of interest. PAFP fusions should be validated in several ways [66]. Sequencing is important to confirm that the fusion is correct, as frameshifts can sometimes arise in the linker, and other random mutations can in principle be introduced. If a good antibody to the protein is available, Western blotting can be used to compare the expression level of the fusion to that of the unmodified protein and to check for truncation or cleavage of the fusion. Finally, a functional assay for the protein of interest can confirm that the fusion protein retains the relevant biological activity. For two-color experiments, similar approaches can be used to generate and validate a second fusion to a standard fluorescent protein (FP) to serve as a marker for replication forks or other cellular sites of interest.

For reproducibility in imaging culture growth, strains should be freshly streaked on solid media containing appropriate antibiotics. After this initial selection step, antibiotics are not necessary for strains containing chromosomal fusions. The morning before imaging, a small scale culture in LB or other rich media can be inoculated from a single colony and grown for several hours until turbid. This “overday” culture can be used to inoculate an “overnight” culture in minimal media. The following day, the overnight culture is used to inoculate a large-scale culture in minimal media for imaging. This imaging culture can be inoculated using a fixed dilution of the overnight culture or to a fixed initial optical density (OD). Growth of the imaging culture can be monitored by recording the OD at 600 nm until it reaches a specified range, generally early exponential phase, at which point a sample can be prepared for imaging. In one standard approach, agarose is dissolved in growth media and deposited between two clean microscope slides or coverslips to cast an agarose pad. Cells are then harvested, concentrated by centrifugation, resuspended, deposited on the pad, and sandwiched between the pad and a clean glass coverslip. Background fluorescence can be minimized by using a high purity agarose and by cleaning the coverslip thoroughly, either by oxygen plasma etching or by sonication in organic solvents and base, often ethanol and 1 M potassium hydroxide. Using minimal media for culture growth also helps to reduce background fluorescence. For experiments focusing on the response of TLS polymerases to DNA damage, DNA damaging agents can be included in the agarose pad, provided they are not fluorescent, or added to the imaging culture. The dose dependence and time evolution of TLS polymerase response to DNA damage can be explored by treating cells with different concentrations of DNA damaging agents for different periods of time before imaging.

A PALM microscope requires at a minimum a near-UV laser, typically a 405 nm diode laser, and a visible laser, typically 561 nm, for photoactivation and excitation of PAFPs, respectively. For two-color experiments in which a separate FP is used to mark replication forks or other sites of interest in the cell, a third laser is needed. Common choices are 488 nm and 514 nm wavelengths, which can excite green fluorescent protein (GFP) and yellow fluorescent protein (YFP) variants. For two-color experiments, a multi-band dichroic filter is used to direct laser excitation to the sample and a multi-band emission filter is used to reject laser light and background fluorescence. Images are generally recorded using a sensitive and fast EMCCD camera. High magnifications of typically 100–150x, corresponding to camera pixel sizes of approximately 100–150 nm, are needed for imaging small bacterial cells. To reduce background fluorescence, it is common to use highly inclined thin illumination, or near-TIRF, in which incident laser light is focused to the back focal plane of a high numerical aperture (NA) objective, instead of epi-illumination [67].

A standard imaging sequence for a PALM movie starts with a pre-bleaching period of 561 nm excitation alone, in which spontaneously activated PAFPs or other sources of background fluorescence are reduced. After this pre-bleaching period, there are two standard imaging sequences. In the first, continuous 405 nm and 561 nm illumination are used to activate and image PAFPs. In the second, 405 nm photoactivation pulses are alternated with periods of 561 nm excitation. In both approaches, the 405 nm laser power is adjusted to ensure that no more than one molecule at a time is activated per cell, and the power may be gradually increased over the course of the movie to maintain a uniform activation rate as molecules are activated and irreversibly photobleached. The continuous activation approach is simpler to implement and avoids breaking up trajectories of activated molecules, whereas the pulsed activation approach avoids background autofluorescence due to near-UV excitation of cells. In two-color imaging experiments with a different marker protein, imaging of the marker can be performed after the pre-bleaching period. Depending on the length of the acquisition and the nature of the marker protein, it may be necessary to image the marker multiple times during the PALM movie.

The choice of integration time depends on the type of analysis to be performed. Short integration times, generally 10–20 ms, are needed to resolve the diffusion of molecules throughout the cell, although higher frame rates may be necessary for smaller proteins that diffuse more rapidly [68, 69]. For selectively resolving bound proteins, such as TLS polymerases recruited to the replication fork, longer integration times can be used to blur out the signal from mobile molecules. Because lower laser powers can be used at these lower frame rates, an advantage of this approach is that it slows photobleaching and thereby enables the measurement of binding dynamics that occur over longer timescales.

Analysis of bacterial cell PALM movies includes two basic steps. The first is the fitting of a shape to the bacterial cell outline, also known as cell segmentation. A number of software packages are available for this task, including Oufti [70] and its predecessor MicrobeTracker [71]. Fluorescence images, either of a cytoplasmic marker or of the cell wall or membrane, can be used for cell segmentation; more commonly in PALM experiments, however, a transmitted light brightfield image is used instead. The second step is the detection and tracking of single molecules and multi-copy foci. Again, there are a number of freely-available software packages implementing different detection and tracking algorithms, including the MATLAB-based package u-track [72, 73]. A common approach is to fit fluorescent spots to a 2D Gaussian approximation of the point spread function. Software packages that can perform one or both of these tasks are available as ImageJ plug-ins, MATLAB suites, or stand-alone applications [74–79].

Once cells have been segmented and spots detected and tracked, a number of specialized analyses can be performed, often using custom-written analysis code. Of particular interest to PALM studies of TLS polymerases, the number of polymerase binding events per cell and the lifetime of these events can be readily determined. By normalization of the cell outline along the long and short axes, an average cellular localization distribution can be generated to reveal the average polymerase localization across many cells, as well as the average localization of replication forks or other replication and repair factors. Finally, in two-color imaging experiments, single-cell colocalization analysis can be performed to determine the distance of polymerase binding sites from a particular cellular position, such as the replication fork. A powerful approach for colocalization measurements is radial distribution function analysis, [80, 81] which normalizes this intra-cell distance distribution by a simulated distribution generated assuming random cellular localization; this approach reveals colocalization, in particular weak or incomplete colocalization, more readily than a simple distance distribution.

#### Cautionary notes

Care must be taken in designing and validating protein fusions to PAFPs, as the addition of an approximately 30 kDa fusion protein can impair activity. When available, structural information and information about interaction domains must be taken into account. The protein terminus to which the PAFP is fused, the length of the linker between the protein and the PAFP, and the particular choice of PAFP can all affect function. In the case of the TLS polymerase Pol IV, we found activity to be impaired for shorter linkers relative to longer linkers, and for the PAFP mMaple3 relative to PAmCherry [62]. In some cases, fusions to one protein terminus may be non-functional, whereas fusions to the other terminus retain activity. For example, only N-terminal fusions have been reported for the *E. coli* sliding clamp processivity factor β, although C-terminal fusions are viable for other components of the replication machinery [82]. In cases where an N-terminal fusion is necessary, it is advisable to remove the associated antibiotic marker, by FLP-FRT recombination [83] or a similar approach, to minimize possible effects on the expression level. Validation of the activity of a PAFP fusion through a functional assay is particularly important for non-essential proteins like TLS polymerases. For an essential gene, successful creation of a PAFP fusion implies that protein function is not completely impaired, although independent validation and assessment of sub-lethal defects is still important.

Careful control experiments are critical for PALM imaging. Excitation of bacterial cells with high intensity laser illumination can lead to significant fluorescence background. In some cases this background is just a diffuse fluorescent haze, but in other cases it manifests as bright fluorescent spots, either mobile or stationary, in the cells or in the agarose pad or coverslip. To assess the possibility of spurious detections of background fluorescence, it is important to image the parent strain of the PAFP fusion under matched imaging conditions. The use of carefully matched imaging conditions is critical; for example, we have observed significantly different levels of background localizations for relatively modest differences in 405 nm photoactivation power. It should also be noted that the level of spurious background localizations varies significantly for different bacterial species [84] and can be affected by the presence of chemical additives like IPTG [81]. In two-color experiments, strains lacking either the PAFP fusion or the marker protein fusion should be imaged to assess possible crosstalk between the channels. Crosstalk in the PAFP channel is most likely from bright, multi-copy marker foci. If such crosstalk proves to be a problem, imaging conditions can be chosen to thoroughly bleach these foci before starting to record the PALM movie.

As for all experiments involving fusion proteins, it is important to be mindful of possible artifacts due to the FP. Many FPs have a tendency to oligomerize, having evolved from naturally multimeric proteins [85]. Several studies have characterized the tendency of popular FP and PAFP variants to aggregate, at least under certain conditions [45, 86, 87]. When possible, highly monomeric FPs should be chosen. For FPs derived from the *Aequorea* jellyfish, like GFP and YFP, variants with the A206K mutation show reduced oligomerization [85]. It is also helpful to verify that key results are independent of PAFP by constructing and imaging the same fusion with a different PAFP. Another useful control experiment is to image the PAFP alone expressed from the same promoter, or at the same chromosomal locus if possible, to confirm that any observed localization is not driven by the behavior of the PAFP itself. If there are concerns about aggregation due to the presence of the fluorescent protein, altering the expression level of the fusion by changing the strength of the promoter or using an inducible promoter can help confirm that the localization behavior and other results are not affected.

Finally, there are a number of approaches that can be used to validate average localization and two-color colocalization analysis. To avoid the loss of information due to averaging a heterogeneous population of cells, localization analysis is sometimes performed only for cells within a certain length range, which serves as a proxy for cell cycle state. Alternatively, localization analysis can be filtered by other cellular parameters, such as the number of replication forks, to look for differences within the population of cells. To ensure that apparent colocalization is robust in two-color experiments, the imaging and analysis can be repeated for a different marker protein. For example, we observed colocalization of Pol IV with replication forks for both a SSB-mYPet marker [62] and a YPet-β marker (unpublished data), as expected. It can also be helpful to perform the colocalization analysis for a PAFP fusion to a protein that is not expected to colocalize with the marker of interest. This analysis can help distinguish similar average localization patterns from true intra-cell colocalization. For example, although a PAmCherry fusion to the DNA-binding protein HU was localized on average in a similar region of the cell as SSB-mYPet foci, we found little intra-cell colocalization as revealed by radial distribution function analysis [62]. To ensure adequate sampling of data for radial distribution function analysis, it is important to generate a random *g(r)* curve for the data set of interest; this random *g(r)* curve should be close to 1 for all *r* values. Large deviations from 1 in the random *g(r)* curve indicate that the data set is too small. Another approach that we have found helpful is to simulate multiple random localization distributions for the data set and to analyze the spread in the calculated *g(r)* curves to ensure that results are robust.

#### Conclusion

Particle-tracking PALM and two-color fluorescence imaging in live bacterial cells are powerful and versatile techniques that are providing new insight into the recruitment and action of TLS polymerases and other DNA replication and repair proteins. Careful control experiments, however, are critical for both the biological and imaging aspects of these assays. To ensure that results are physiologically relevant and not artifacts driven by the presence of the FP, it is important to confirm the functionality of PAFP fusions. As for all single-molecule imaging experiments, it is also necessary to minimize sources of background fluorescence and to validate analysis methods.

### Tracking-PALM: a direct single-molecule imaging method to study DNA repair in living bacteria

#### Overview

New *in vivo* single-molecule imaging and tracking methods that break the diffraction limit are transforming our understanding of complex biological processes, their structural organization and their dynamics inside bacterial cells. One of the most successful of such methods relies on the combination of single-particle tracking (SPT) [88] with PALM [63], a popular localization-based super-resolution imaging technique. This method, also known as “tracking PALM” [43, 68, 89, 90] has also been used to study DNA damage and repair at the single-molecule level inside living bacteria [19, 91, 92].

Here, we review practical aspects related with the use of tracking PALM for studying DNA repair in live *E. coli* cells; most considerations are relevant to the study of other DNA-binding proteins inside a variety of microbial cells, as well as to other cytoplasmic proteins and membrane proteins.

#### Description of method

Single-molecule tracking experiments typically rely on collecting wide-field images of cells containing molecules (proteins, DNAs, lipids, etc.) labeled with fluorescent probes. In the case of proteins, those probes are typically auto-fluorescent proteins (such as GFP derivatives) or organic fluorophores (introduced using Halo- or SNAP-tagging) [93, 94], with the first approach being more popular due to the specificity and simplicity of the genetic labeling; in contrast, Halo/SNAP-based labeling requires cloning a Halo/SNAP protein fusion (containing a Halo/SNAP domain, which has a size comparable to GFP) at one of the protein termini, the addition of an appropriate fluorescent substrate to the cell containing the fusions, and a subsequent wash to remove any unincorporated substrate.

Here, we will describe the workflow for the use of photoactivatible GFP (PAFP) derivatives (such as photoactivatable mCherry, i.e., PAmCherry) [44], which is a cornerstone of PALM. Use of a photoactivatable GFP derivative allows proteins of essentially unlimited copy number (>100,000) to be examined, making the activation-based approach much more general. In contrast, if the GFP-protein fusion is fully fluorescent after folding and fluorophore maturation is complete, it is only possible to work with proteins of very low copy number (1-5 molecules per cell); another approach is to photobleach many of the proteins to arrive in the 1-5 molecule-per-cell regime, but this also lowers the statistics obtained per single cell.

A prerequisite of tracking PALM is typically the construction of a fusion of the protein of interest with the PAFP, connected by a small amino acid linker. The choice of linker can impact protein function; in most cases, the linker is chosen from a wide range of sequences that vary in length and flexibility (reviewed in Chen 2013) [95]. The linker that has shown most success with PAmCherry is a flexible linker comprised of 11 amino acids (SAGSAAGSGEF), rich in small or hydrophilic amino acids such as Gly and Ser [96]. The PAFP can be inserted at either termini or as an internal fusion. However, the C-terminal fusion is the most widely used as it is easier to obtain chromosomally than either the N-terminal or internal fusions. If the C-terminal fusion compromises the protein activity, more challenging protein fusion positions can be explored. Regardless of the insertion position, protein function should be assayed after every fusion. Unsuccessful fusions of essential genes with PAFP are easily screened by the absence of viable colonies after antibiotic selection on LB-agar plates. A loss or reduction of function, on the other hand, can be screened by comparing the fusion strain's growth rate to that of the wild-type strain; testing the cellular morphology under the microscope (i.e., cell size and shape); and screening for process-specific phenotypes, e.g., the ability to respond to DNA damage [19].

**Preparation of bacteria for imaging on agarose pads.** To study DNA repair, multiple methods can be undertaken depending on the nature of the DNA repair mechanism studied [19, 62]. In general, cells are pre-cultured by inoculating a single colony in LB at 37°C for 4-5 hours, or in M9 supplemented with 0.4% glucose for up to 8 hours. Cultures are then diluted 1:1,000 in M9 supplied with 0.2% glucose or glycerol overnight at 37°C. The following day, the cells are diluted in fresh M9 media until they reach the early exponential growth phase with OD_600_ 0.1-0.2. Suitable DNA-damaging agents for a specific DNA repair pathway are then either incubated with the culture for up to an hour prior to PALM imaging [62], or are added directly into the agarose pad preparation [19]. Cells are then spun down and immobilized on the lower side of an agarose pad sandwiched between two coverslips. Pads of 1% agarose are made by mixing low-fluorescence 2% agarose in dH_2_O with 2x M9 culture medium and pipetted onto the coverslip. In preparation for single molecule fluorescence microscopy, the coverslips should be burned or sonicated to remove any contaminants that may contribute to the auto-fluorescence background.

**PALM imaging system.** Experiments are performed on a single-molecule fluorescence microscope, with laser illumination used for photoactivation and excitation (e.g. λ_activation_∼405-nm, λ_excitation_∼561-nm for PAmCherry). The high sensitivity and temporal resolution typically required (≤ 15 ms/frame) make EMCCD and sCMOS cameras popular choices for image-acquisition systems.

The mode of illumination is chosen to match the region of the sample being studied. In TIRF microscopy, excitation is generated by an evanescent field which penetrates 100-200-nm into the sample, limiting the region of study to a shallow depth, but very effectively suppressing background fluorescence. Epifluorescence illumination excites molecules throughout the sample, generating significantly more noise. Highly-inclined Laminated optical sheet (HiLo) microscopy is common in PALM experiments as it offers an intermediate between these two extremes, illuminating the majority of a bacterial cell's volume, whilst providing excellent signal-to-background noise characteristics.

**Figure 7:**
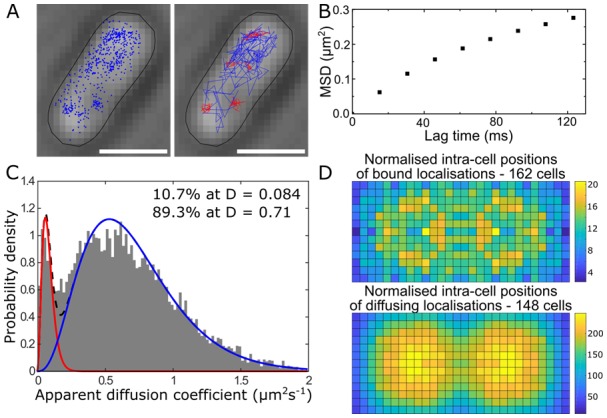
FIGURE 7: PALM imaging. Tracking PALM experiment with DNA polymerase I-PAmCherry fusions in *E. coli*, performed 30 min following treatment with 100 mM MMS. **(A)** Bright-field image of a single *E. coli* cell superimposed with the detected cell boundary mesh, molecular localizations (left), and compiled molecular tracks (right). Scale bars, 1 µm. **(B)** Mean MSD analysis obtained for 2,048 molecular tracks. **(C)** Apparent diffusion coefficient histogram from 2,048 molecules, calculated from MSDs; the inset numbers indicate the proportion of molecules in each population. **(D)** Heat maps of molecular localizations for the bound (top), and freely-diffusing (bottom) populations.

**Acquisition and reduction of PALM data.** The intensity of the photoactivation light is adjusted such that there is a maximum of approximately one fluorescent particle per cell at any given time. A typical tracking PALM experiment lasting 2.5 minutes, collects single-molecule tracks from the localizations of 400 molecules per cell, arising from a typical field of view containing 10-100 cells. A total of 10,000 frames are acquired during a single experiment. Initially, each frame is processed separately to extract particle positions (**Figure 7A**, left). Tracks are then identified by linking particle localizations, both spatially and temporally (**Figure 7A**, right). The localization process identifies point spread functions (PSFs) for each particle within a frame using elliptical Gaussian fitting. Cell tracks are reconstructed by searching for localizations in neighboring frames that fall within a maximum spatial distance; this threshold also depends upon the frame rate. Cell segmentation is performed using an algorithm that determines the edges of cells present in a bright-field or phase-contrast image taken immediately before the experiment, extracting a set of meshes which define the boundary of each cell within the field of view. The segmentation process, which assigns the localizations to individual cells, may be implemented either before or after the localization and tracking procedures.

**Molecular mobility.** The localizations and reconstructed tracks contain a great deal of information about the underlying processes studied. First, the number of localizations per cell provides an indication of the approximate copy number of the labeled protein. This can be used to detect differential expression of the protein of interest by comparing the number of localizations in the original and mutated strains.

Further, the traces offer ample information to characterize different types of diffusion. A popular method for the classification of diffusion modes is based on the MSD obtained from the trajectories of individual molecules. The gradient of the plot of MSD over a range of interval times gives the mobility of the molecular species being studied. A linear MSD plot indicates free diffusion, unbounded by a nucleoid or cell periphery, such that the particles obey Brownian motion with random movements originating from collisions with other molecules. It is common for acquired MSD curves to be non-linear, such that the particles exhibit anomalous diffusion. Anomalous diffusion is sub-categorized into either sub-diffusion, where diffusion is either confined geometrically, or limited by molecular interactions; or super-diffusion, where movement is governed by an external process or structure, such as active molecular transport, or cellular tracks. Immobile molecules exhibit the extreme case of sub-diffusion, however, the resulting MSD is non-zero due to contributions from the localization error (which generate apparent movements in the 1–40 nm range), uncorrected microscope drift, cell motion, and cell growth.

Further analysis of the MSD enables separate diffusion behaviors to be identified. Uphoff *et al.* [19] investigated MSDs of DNA polymerase I (Pol), and DNA ligase (Lig) following DNA damage by methyl methanesulfonate (MMS). We illustrate this method in **Figure 7B** with MSD analysis of Pol molecules. The apparent diffusion constant, D*, for each molecule was extracted from the MSD via the relation D* = MSD/(4Δt) − σ_loc_^2^/Δt, where Δt is the interval time between localizations, and σ_loc_ is the localization precision. Diffusion constants for each track were then compiled into a histogram (**Figure 7C**), yielding two distinct diffusing populations which represent the bound and diffusing populations of Pol following MMS treatment. As Pol binds to DNA during the repair process, its kinetics change from the high diffusion constant of the Pol molecules during the search process as it diffuses through the nucleoid, to the lower diffusion constant associated with pol binding to DNA [19].

In addition to the large aggregated diffusion data generated by tracking PALM, the technique also allows all individual tracks to be interrogated separately. Uphoff *et al.* used this approach to study individual, complete Pol tracks showing the sequence of diffusion behaviors involved in DNA repair: searching for DNA repair locations, binding and unbinding to the chromosome, followed by a return to free diffusion through the nucleoid [19]. In doing so, they were able to capture timescales for the full search, and repair processes, identifying a mean repair time of 2.1 seconds for Pol.

Furthermore the boundary of particle tracks also reports on a particle's confining geometry, for example, nucleoid-associated proteins such as heat unstable protein (HU) effectively trace out the extent of the nucleoid [97].

Analysis of localizations using clustering algorithms, such as Density Based Spatial Clustering of Applications with Noise (DBSCAN), provides unbiased, quantitative identification of molecular clusters [98], allowing theories regarding the dynamic clustering of protein machinery, such as transcription factories [99], to be investigated in detail. Such a study has already provided early evidence of the absence of an equivalent system in DNA repair [19].

#### Cautionary notes

**Labeling.** Functionality of the target protein should not be compromised by the PAFP to which it is fused. It is thus important to avoid disrupting functional domains or protein folding. To achieve this, multiple steps can be taken towards a successful fusion. Fusion sites in regions that have previously been documented as crucial for interaction with other proteins or DNA should be avoided. If unavoidable, linkers with varying length and rigidity may be tested. It is often possible to insert a flexible linker that will permit the protein to carry on its function while harboring the PAFP at a distance [62]. This method might also help in situations where the PAFP is larger than the protein of interest. Adding a long flexible linker might separate the protein and the PAFP far enough that both proteins fold correctly and independently.

Species of PAFPs vary in their tendency to dimerize. Some protein fusions may cause synthetic aggregation which will impact upon protein tracking, and by extension the validity of the acquired data [100]. It is crucial to perform control measurements to verify that a protein fusion is fully functional, does not aggregate or multimerize significantly, and gives biologically relevant information, prior to tracking PALM experiments. In the case of DNA repair, it is important to run microbiological tests, such as minimum inhibitory concentration tests, to establish whether the protein fusion strains have retained a similar capacity to survive a dose of the DNA damaging agent as does the WT. A final, crucial test for proteins involved in DNA repair is a clearly observed decrease in diffusion constant upon induction of DNA damage.

**Light can be perturbative.** When studying DNA repair, conditions leading to stress at both the molecular and cellular levels should be minimized. Tracking PALM often relies on photoactivation performed with peak wavelengths in the UV or 405-nm (as used for PAmCherry). Long exposure to the 405-nm laser can cause temporary cessation of growth [19], and at high doses also DNA damage and cell death, introducing errors to the DNA repair experiment.

**Quantitative aspects.** Care must be taken whenever using the number of localizations to estimate the copy number of proteins, as many factors contribute to miscounting; incomplete maturation of fluorescent proteins, fast bleaching, motion blur, and incomplete illumination throughout the cell volume may all contribute to under-counting, while blinking and the presence of fluorescent contaminants may contribute to over-counting. In addition to this, using poorly chosen parameters during the tracking analysis can contribute to either under- or over-counting.

Blinking, in particular, presents significant problems if not handled effectively [101]. A single fluorescent probe undergoing blinking will appear to many tracking algorithms as multiple tracks with very high spatial, and temporal, proximity. This would be interpreted incorrectly as multiple independent tracks exhibiting clustering. To address this specific issue, a “memory parameter” is used, which specifies the number of frames for which the fluorophore can exist in its “off” state, and still be linked by the analysis software to the original track. Careful selection of this parameter is important; too high, and the probability of connecting truly independent tracks increases to unacceptable levels, leading to under-counting; too low, and the memory parameter will be ineffective. Parameter selection is a balance, and little flexibility in one parameter may often be offset by adjusting another. Using the present example, if a very large memory parameter is required, its effects may be mitigated by reducing the photoactivation intensity, and increasing the total experiment time, thus separating tracks temporally. While selection of the fluorescent probe may limit blinking, it is also often very useful to characterize its blinking properties in the same context in which it is being used in order to set experimental parameters; for example, a probe may exhibit different blinking characteristics in live, and in fixed cells.

**Photobleaching limitations.** Track length, observation span, and localization precision in tracking PALM experiments are limited by the photobleaching rate, which in turn is dependent upon the fluorophore's photon budget, i.e. the number of photos emitted before moving irreversibly to the non-fluorescent state. Organic fluorophores have been shown to exhibit significantly higher photostability, with observable timescales on the order of a few seconds, enabling the study of dynamic biological processes previously inaccessible due to their long duration. Recent developments in Halo-Tag, and SNAP-tag technology have enabled such organic fluorophores to be attached to target proteins in live cells. Both methods involve forming a fusion protein consisting of the protein of interest, and a linker (the Halo-Tag or SNAP-tag) which accepts a ligand such as an organic fluorophore. For Halo/SNAP ligands unable to pass directly through the cell membrane, the use of electroporation for the reliable incorporation of essentially any fluorescent probe directly into bacterial cells has been demonstrated [102]; this allows the incorporation of a wide range of fluorescent ligands using Halo-Tag and SNAP-tag technologies. Furthermore, recent developments in highly stable photoactivatable organic fluorophores [103, 104] will enable tracking PALM measurements to be performed *in vivo* over longer timescales than with existing PAFPs.

**Fitting of distributions.** Accurate fitting of the diffusion histogram (**Figure 7C**) is essential, as errors from the fitting procedure may lead to radically different interpretations of the diffusive species present, and by extension the underlying biological processes. A good fitting of distributions minimizes residuals of a gamma fit to the data. For example, Stracy *et al.* explored different fitting models for the diffusion coefficient histogram of UvrB, a protein essential for NER, and obtained three diffusive species: one bound and two mobile populations [96]. Similar complexity was also observed for the *lac* repressor [81]. The goodness of fit can also be improved by varying experimental parameters that enhance unclear populations; e.g., poor fitting to bound populations may be improved by increasing DNA damage via exposure to higher concentrations of MMS. The Stracy *et al.* work used deletion mutants and over-expression of UvrB to show that UvrB availability only mildly influenced UvrA-binding times, both in the presence and absence of DNA damage [96]. Parallel control experiments are vital to make putative fitting more robust and convincing.

**Low copy numbers.** The presence of auto-fluorescent particles presents a greater problem for the tracking of low-copy number proteins than for high-copy numbers, as the small quantity of contaminants that form tracks make up a larger proportion of the total recorded data. A key challenge for studying molecules with low-copy numbers is in acquiring enough tracks to obtain reliable statistics whilst suppressing noise. Garza de Leon *et al.* tracked single *lac* repressor (LacI) molecules over-expressed in a plasmid to gain a larger statistical sample, and followed the mobility and subcellular distribution of LacI [81]. Studying the over-expression profile may serve as a reference to characterize the more complex landscape that occurs under natural conditions with low copy numbers (40-80 for LacI). Further, it is possible to characterize the contribution from auto-fluorescence contaminants by performing tracking PALM with a control sample that does not express the PAFP fusions.

**Fixed cells vs live cells.** Super-resolution imaging can be performed with both fixed and live cells. In fixed cells, all of the molecules are stationary, allowing for precise localization of particles in cells fixed rapidly at specific stages of the growth cycle. For example, Endesfelder *et al.* investigated the spatial distribution of RNAPs in fixed *E. coli,* and characterized large and small RNAP clusters in rich and minimal growth media [98]. In complementary imaging experiments of live cells, Stracy *et al.* obtained two species of RNAP with different diffusive profiles: mobile RNAPs searching for promoter targets throughout chromosome, and bound RNAPs engaged in active transcription around the periphery of the nucleoid [97]. Experiments in fixed cells permit more controllable capture of profiles at specific treatment points, while live-cell imaging report on dynamic processes, such as the motion of RNAP and its clusters [97]. It is often useful to obtain complementary PALM data from both fixed and live cells to provide a more complete picture of a complex process such as transcription and DNA repair.

**Species can interconvert between diffusive states.** DNA-associated proteins, such as those involved in DNA repair, are often assumed to transition dynamically between two diffusive states, representing diffusing proteins and those bound to the chromosome. While tracking-PALM enables us to precisely observe the diffusion of individual molecules in the absence of ensemble averaging, it is important to ensure that incorrect assumptions of the number of states do not interfere with the fitting process. To achieve this, an objective and quantitative approach based on hidden Markov models (HMMs) is often applied to identify the true number of states of the system [105]. Exploring dynamic transitions between diffusive states in this way leads to a more accurate determination of the diffusion constants, and may provide insight into previously unknown kinetics of the protein of interest.

**Statistics.** Reliable statistics are important for tracking-PALM to build an accurate diffusion coefficient histogram, and heat maps of mean spatial distributions, for a given molecular species. However, meaningful statistics often require cautious selection of cells with homogeneous profiles; it is essential to pool together cells of comparable dimensions and similar physiological states. In practice, large quantities of localization data from multiple cells are combined and visualized by first sorting cells according to cell length (as an indicator of cell age), and positions are normalized by cell length and width, such that all localizations can be displayed on a single heatmap (see **Figure 7D** for an example). This workflow provides a powerful and rapid approach to understanding the mean spatial distribution of all molecules for a given physiological state.

**Two-color PALM.** Two-color PALM measurements are advantageous when examining molecular partner interactions or co-localizations. However, two-color imaging in live cells is still challenging since molecular partners are not visualized at the same time, which raises the problem of accurate real-time interactions since all of the components, such as the nucleoid or membrane, are moving inside cells.

Two-color imaging by combination of photoactivatable fluorophores with non-photoactivatable fluorescence markers is more feasible, as the second color can be imaged first with a short period of time to obtain subcellular localizations, and PALM imaging with photoactivatable fluorophores can be acquired afterwards. Garza de Leon *et al.* observed the co-localization of a multi-probe fluorescent genomic marker (specifically, a fluorescent repressor operator system, FROS) with PAmCherry-labeled LacI clusters [81]. In another example, Stracy *et al.* imaged the nucleoid geometry using staining with the SYTOX DNA stain, which closely matches the distribution of mobile RNAPs (which was probed in a different emission channel) [97].

#### Conclusion

Tracking-PALM is increasingly being used as a powerful tool to elucidate protein-DNA interactions at the single-molecule level, revealing important dynamics previously hidden by ensemble averaging (e.g. in FRAP experiments; [106, 107]); the method has already been used to follow single-molecule trajectories of DNA-repair machinery operating on the bacterial chromosome, revealing the kinetics of DNA-repair processes.

The technique has enormous potential for answering a wide range of biological questions involving the movements of single molecules beyond the diffraction limit. Rapid advances currently taking place in labeling methods such as Halo/SNAP-tagging, and highly stable photoactivatable organic fluorophores, are allowing tracking PALM to be applied to a much broader range of contexts as a general method for the study of single-molecule interactions *in vivo*.

Substantial information may be derived from the initial localizations. Linking localizations into tracks provides a history of a molecule's movements, from which important kinetic properties of the molecule are extracted. MSD analysis reveals the diffusion constants that help characterize the observed motion; any inter-conversion between diffusive states leads to insights about the molecule's behavior. Tracking-PALM data can be further analyzed to identify the clustering of molecules, and by implementation of two-color PALM, to detect co-localization with other cellular components. This vast quantity and variety of information provides the means to directly test theories such as DNA-repair mechanisms, and transcription factories, inside living cells.

However, caution must be taken at many points of the experiment, data reduction, and analysis. Interpretation of tracking PALM results can be strongly affected by sources of error during labeling, image acquisition, data reduction, and MSD analysis. Furthermore, experiments should always be performed in the presence of many biological controls.

Future improvements to the technique are likely to come from new fluorophores; complex illumination schemes that adjust the timescale of detection to processes of slow timescales (1-10 min); facile integration into microfluidic systems; combination with similar methods, such as FRET; and better data analytics (e.g., improved HMMs to study diffusion states, and crowded-field algorithms for dense localizations).

## BIOCHEMICAL ASSAYS FOR STUDYING HOMOLOGOUS RECOMBINATION-MEDIATED DNA REPAIR

HR, a template dependent DNA break repair mechanism, plays important roles in DNA damage repair, recovery of injured replication forks, telomere maintenance and repair of meiotic Spo11-dependent DSBs. The HR machinery, centered around Rad51 and Dmc1 recombinase catalyzed homology search and strand exchange, also entails several other key enzymatic activities, e.g. DNA nuclease, DNA helicase, DNA polymerase and topoisomerase, to ensure a faith repair. The action mechanism of HR is mostly revealed by studies of DSB repair as a model system. Specifically, HR mediated DSB repair is initiated by the generation of 3′-ssDNA via digestion of the 5′ strand, which is fulfilled by several mechanisms including 1) Mre11-Rad50-Xrs2-Sae2 ensemble which digest duplex DNA via a combining action of an endonuclease activity and a 3′ exonuclease activity [108]; 2) Exo1-catalyzed exonucleolytic digestion; 3) Sgs1 catalyzed duplex unwinding and its coupled 5′ ssDNA digestion by Dna2 nuclease [109, 110]. The ssDNA generated by DNA end resection is first coated by ssDNA binding protein, RPA, which triggers ATR/Mec1 dependent DNA damage checkpoint cascade [111]. Next, mediator protein aided Rad51 nucleoprotein filament formation onto RPA coated ssDNA enables homology search and subsequent strand exchange reaction. The resulted displacement loop (D-loop) structure entails a primer/template joint of the invading 3′ end, which triggers DNA repair synthesis by Polδ in a manner that is stimulated by Pif1 via a D-loop migration mechanism [112–114]. Extension of the invading 3′ end generates DNA sequence complementary to the 3′ ssDNA at the opposing DSB end, thereby allows capturing of the second DSB end by either the newly synthesized strand or the strand it displaced, two outcomes that channel the repair into synthesis-dependent strand annealing (SDSA) pathway and double strand break repair (DSBR) pathway respectively. The choice between SDSA and DSBR is regulated by Mph1 and Srs2 helicases, which dismantle the D-loop structure and free the invading 3′ strand for its annealing to the opposing DSB end [115, 116]. Capturing the non-invading 3′ strand by the D-loop leads to the formation of double Holliday junction (dHJ) structure. In vegetative growing cells, dHJ is predominantly processed by Sgs1-catalyzed branch migration and the subsequent decatenation by Top3-Rmi1 complex, a mechanism termed as dHJ dissolution, which completes the repair without the formation of crossovers [117, 118]. Alternatively, the dHJ structure may be processed by a class of structure-specific endonuclease, namely the resolvase (e.g. Yen1) [119], via a mechanism termed as resolution. Different from the dissolution mechanism, the resolution pathway may introduce crossover, thus less favored in vegetatively growing cells. Before full maturation of dHJ, the D-loop with second end captured, may be processed by the structure-specific endonuclease Mus81/Mms4 as a salvage means, which, in meiosis, plays an important role in clearing the complex joint molecules involving multiple chromatids [120–122]. Notably, Srs2 is also able to salvage the non-productive Rad51 filament by clearing Rad51 from ssDNA, which allows the DSB repair to be completed via alternative means, e.g. single-strand annealing [123, 124]. Herein, we described a series of biochemical assays on studying Rad51 recombinase, DNA polymerase, DNA helicase and structure-specific DNA nuclease, which help to dissect the mechanistic details of the HR machinery (**Box2**).

BOX 2: **BIOCHEMICAL ASSAYS FOR STUDYING HOMOLOGOUS RECOMBINATION MEDIATED DNA REPAIR**

**Recombinase filament assembly |** Assays for assembly of the presynaptic filament in homologous recombination with Rad51 or Dmc1 recombinase. Both an endonuclease protection assay and biotinylated-ssDNA bead assay are presented.

**DNA repair synthesis following D-loop formation |** Assays for repair synthesis using the D-loop as a primer are described. The activity of DNA polymerase δ and its stimulation by the DNA helicase Pif1 are discussed.

**DNA helicases and function in processing homologous recombination intermediates |** Assays for DNA helicase substrates and their roles in completing homologous recombination are presented.

**Fluorescence-based assays for structure-selective endonucleases |** Structure-specific endonucleases are key enzymes in processing DNA joint molecules that are intermediates in the recombination process. Here assays for different types of joint molecules are discussed. Both gel-based and FRET-based assays are presented.

### Biochemical analyses of recombinase filament assembly

Assembly of recombinases on ssDNA, namely presynaptic filament, is a critical step for regulating recombination [125, 126]. In most eukaryotic cells, there are two evolutionally conserved RecA family recombinases, Rad51 and Dmc1. Rad51 is a prerequisite for mitotic recombination; in contrast, Dmc1 is specific for meiotic recombination [127, 128]. Despite their distinct expression distribution, they do share similar biochemical characteristics. Both recombinases possess a conserved Walker A motif that binds and hydrolyzes ATP. Importantly, Rad51 and Dmc1 nucleate on ssDNA to form a helical protein filament that stretches B-form DNA into 1.5 fold of its original length. ATP is required for the assembly of a functional Rad51/Dmc1 nucleoprotein filament, and ATP hydrolysis leads to the disassembly [129, 130]. It has been well documented that calcium ions and recombinase-associated partners regulate the assembly and disassembly of presynaptic filaments [131, 132]. Enhancement of filament formation greatly stimulates Rad51/Dmc1-mediated homologous DNA pairing and strand exchange activity [129, 130]. In budding yeast, *Saccharomyces cerevisiae*, the assembly of the Rad51 filament is tightly regulated by its interacting partners, Srs2 helicase and Rad55-Rad57 complex. Srs2 helicase physically interacts with Rad51 and translocates along ssDNA to remove the roadblock Rad51. Of note, the physical protein-protein interaction of Rad51 and Srs2 strengthens the motor activity of translocase. In contrast to filament disassembly, Rad55-Rad57 complex stabilizes Rad51 filament [133, 134]. As such, assembly or disassembly of presynaptic filament reflects the efficiency of recombination in response to its physiological needs. Here, we describe the biochemical methods to determine the stability of presynaptic filament.

**Figure 8:**
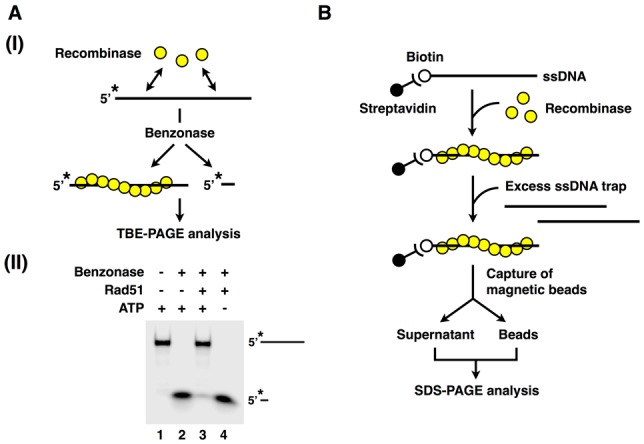
FIGURE 8: Endonuclease protection assay. **(A)** A schematic representation of endonuclease protection assay in (**i**). Assembly of *S. cerevisiae* Rad51 filaments was analyzed with ATP or no nucleotide as cofactor in (**ii**). **(B)** Schematic of biotinylated-ssDNA bead-based assay.

Endonuclease protection assay (**Figure 8A** (i)) and biotinylated-ssDNA bead-based method (**Figure 8B**) are relatively convenient approaches to monitor the assembly of Rad51/Dmc1 nucleoprotein filament.

#### Endonuclease protection assay

**Preparing radiolabeled ssDNA substrate.** Materials: PAGE purified 80-mer ssDNA: 5′TTATGTTCATTTTTTATATCCTTTACTTTATTTTCTCTGTTTATTCATTTACTTATTGTATTATCCTTATCTTATTTA, γ-^32^P-ATP (10 mCi/ml, PerkinElmer), T4 polynucleotide kinase (T4 PNK, New England Biolabs) and PNK 10X buffer, Benzonase (Sigma-Aldrich), Micro Bio-Spin^TM^ 6 column (Bio-Rad). Buffer A (35 mM Tris-HCl, pH 7.5, 1 mM DTT, 1 mM ATP, 2.5 mM MgCl_2_, 50 mM KCl, and 100 ng/μl BSA), TBE buffer (89 mM Tris, pH 8, 89 mM borate, and 2 mM EDTA).

A gel purified 80-mer oligonucleotide (6 μM) is mixed with γ-^32^P-ATP (40 μCi) and T4 PNK (30 units) in 60 μl PNK reaction buffer at 37°C for 1 h. Heat-inactivate the reaction at 70°C for 10 min. The free unincorporated γ-^32^P-ATP nucleotides are separated from radiolabeled ssDNA by a size exclusion Spin 6 column. The amount of radiolabeled ssDNA is then quantified by spectrophotometer. Note that, alternatively, the radiolabeled ssDNA can be replaced by fluorescence-labeled ssDNA as substrates.

**Endonuclease enzyme challenge.** Typically, a 5′-^32^P-labeled 80-mer ssDNA (3 μM nucleotides) is incubated with Rad51 (1 μM) in 8 μl buffer A at 37°C for 5 min to assemble the nucleoprotein filament. Then, the filament stability is challenged by adding endonuclease Benzonase (5 units) into the reaction mixture to a 10 μl final volume. After 10 min of incubation at 37°C, the reaction mixtures are terminated with a 2.5 μl stop solution containing 240 mM EDTA, 0.2% SDS, and proteinase K (0.32 mg/ml) and incubated at 37°C for 15 min. The samples are subjected to electrophoresis in 10% polyacrylamide gel with TBE buffer. Then the gel is dried and the DNA species are revealed and quantified by phosphorimaging analysis (Bio-Rad). Alternatively, the phosphorimaging system can be replaced by the conventional X-ray film processing system. If Rad51 forms a stable filament on ssDNA, then the radiolabeled ssDNA will be protected against the endonuclease digestion (**Figure 8A**(ii)). Quantification of full-length undigested radiolabeled ssDNA indicates the stability of the presynaptic filament. Note that to analyze how Rad51-associated partners contribute to filament stability, these accessory factors can be added to the reaction after assembly of the presynaptic filament.

#### Biotinylated-ssDNA bead-based method

This method has been successfully applied to determine the stability of human RAD51 presynaptic filament [135].

Materials: 5′-biotinylated 83-mer oligo dT, Streptavidin magnetic particles (Roche)

**Linking biotinylated-ssDNA to streptavidin magnetic beads.** To prepare magnetic beads containing ssDNA, 5′-biotinylated 83-mer oligo dT is immobilized on streptavidin-coated magnetic beads according to manufacturer instructions.

**Determining the remaining bound RAD51 proteins on ssDNA-beads.** To assemble the presynaptic filament, magnetic beads containing biotinylated 83-mer oligo dT are incubated with RAD51 with near 1:3 of RAD51: nucleotide molar ratio in buffer A containing 0.1 mM ATP and 1 mM MgCl_2_ at 37**°**C for 5 min. Then, a final 20 μl reaction is completed by adding ten molar excess of the non-biotinylated ssDNA as a competitor to trap free RAD51. After a 10 min incubation with gentle tapping at 37**°**C, the beads are captured by the Magnetic Particle Separator. Note that the supernatant is set aside for later analysis. After the beads are washed quickly with 20 μl buffer A, RAD51 proteins are eluted with 20 μl 2% SDS. Then, the supernatant and eluate are analyzed by SDS-PAGE to determine their protein amounts. If the majority of RAD51 stays in eluate rather than in supernatant, this indicates that RAD51 forms a stable filament. Note that to analyze how RAD51-associated partners contribute to filament stability, these accessory factors can be added to the reaction after assembly of the RAD51 presynaptic filament.

Both assays described above allow us to monitor the equilibrium state of filament stability under various conditions. In contrast to the bead-based method, the endonuclease assay can evaluate whether Rad51/Dmc1 forms a filamentous structure on ssDNA, because Rad51/Dmc1 binds ssDNA but can't protect ssDNA upon endonuclease challenge in the condition without the presence of ATP. However, protein purity is extremely important for the endonuclease protection assay because any nuclease contamination will affect the analysis readout. Beyond these assays, many methods have been used to monitor presynaptic filament stability. For example, the single-molecule based approach has been applied to determine the nucleation and extension rates of filament assembly [136]. The apparent K_on_ and K_off_ rates can be extracted from the single-molecule analysis in real-time, but not from the bulk assays as we mentioned above. It has been well documented that the number of filaments can be measured by electron microscopy (EM) with negative staining [137]. Although the detailed filament structures, including helical pitches, could be observed, the electronic microscope-based analysis is more technically demanding and relies on the EM instrument in contrast to biochemical analysis. Finally, similar enzyme and bead-based approaches have been applied to measure the assembly of recombinases on dsDNA. For example, the restriction enzyme protection assay has been used to address human RAD51 stability on dsDNA [138], and the biotinylated-dsDNA bead-based method has been used to address how translocases Rad54 and Rdh54 strip off Rad51 or Dmc1 from duplex DNA respectively [139, 140].

Assembly of the presynaptic filament is a critical and initial step for subsequently engaging duplex DNA, searching for homology, and exchanging DNA. Many accessory factors are evolutionally conserved and interact with recombinases to regulate the formation of presynaptic filaments. As such, the *in vitro* methods used for monitoring the filament stability under various biochemical conditions are in demand. The two approaches described here, endonuclease protection and biotinylated-ssDNA bead-based assays, are convenient to be adapted for monitoring the stability of nucleoprotein filaments.

### Assaying DNA repair synthesis by DNA polymerase δ and its stimulation by Pif1 helicase

During DNA double-strand break repair by HR, following the formation of the D-loop, the next critical step is to synthesize new DNA using the invading strand within the D-loop structure as the primer. This serves to replenish the DNA sequence lost during the initial resection step of HR, and the extent of DNA synthesis also helps determine whether crossover or non-crossover recombinants are made. Reconstitution of the “repair synthesis” reaction with purified budding yeast proteins has provided a valuable experimental framework that allows us to interrogate roles of various HR factors in this reaction. This section provides an overview of the biochemical methods for the reconstitution and analysis of the repair synthesis products. Key factors that promote or restrict the extent of repair DNA synthesis are described.

#### The D-loop reaction

The first step in DNA DSBR by HR entails the nucleolytic resection of the 5′ terminated strand at a break end to yield a 3′ ssDNA tail. In mitotic cells, Rad51 is the recombinase enzyme that catalyzes assimilation of the 3′-tailed ssDNA into a homologous duplex donor to form the displacement loop, or D-loop. For the catalysis of D-loop formation, Rad51 must first assemble into a filamentous polymer, called the presynaptic filament, on the ssDNA substrate [129]. Presynaptic filament assembly is an ATP dependent process [138, 141]. Several accessory factors that can enhance the D-loop forming activity of the presynaptic filament have been identified, and these include Rad54, Rdh54, RAD51AP1-UAF1 complex and the BRCA1-BARD1 complex [142–147].

**Figure 9:**
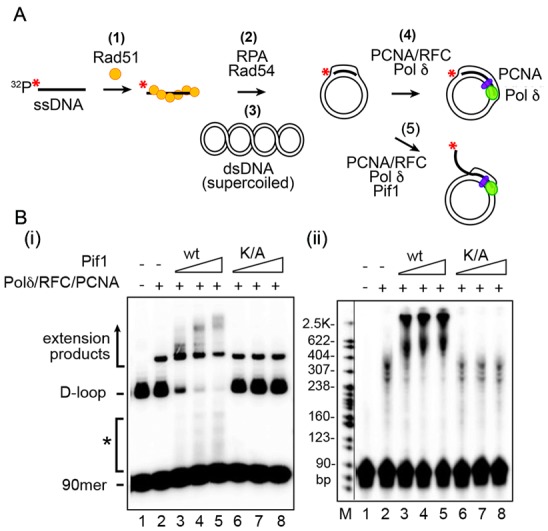
FIGURE 9: Effect of Pif1 DNA helicase on Pol δ-mediated DNA synthesis. **(A)** Reaction scheme to examine the effect of *S. cerevisiae* Pif1 helicase on Pol δ-mediated DNA synthesis. **(B)** Reactions conducted with PCNA-RFC-Pol δ in conjunction with Pif1 or the helicase-dead mutant pif1 K264A (K/A). Analysis was by **(i)** native gel electrophoresis and **(ii)** denaturing gel electrophoresis (7M urea, polyacrylamide) [114]. The asterisk identifies extended invading ssDNA species that had been released from the D-loop by Pif1. (The gel image is taken from [114]).

Reconstitution of the D-loop reaction typically utilizes a ^32^P-labeled ssDNA oligonucleotide to assemble the presynaptic filament and a supercoiled dsDNA plasmid containing a homologous target sequence to enable D-loop formation (**Figure 9A** (1)). Experimentally, Rad51 is incubated with the radiolabeled oligonucleotide at a Rad51:DNA ratio of 1:3 nucleotides in the presence of ATP and an ATP-regenerating system [148]. It should be noted that ATP is also required for the activity of Rad54 and the RFC complex at later steps. Oligonucleotides between 80 to 200 nucleotides in length have been used to make D-loops [149–151]. The single-stranded DNA binding protein RPA helps ensure the assembly of a contiguous presynaptic filament by removing secondary structure in the DNA [152] and it also stimulates DNA synthesis by sequestering ssDNA displaced from the duplex target as synthesis ensues [114, 153, 154]. D-loop formation is greatly stimulated by the addition of Rad54 (**Figure 9A** (2)), which is a member of the Swi2/Snf2 family of DNA translocases [142, 155, 156] and remodels the structure of the dsDNA template to facilitate DNA joint formation [157]. Moreover, by dislodging Rad51 from the nascent DNA joint, Rad54 enhances access of DNA polymerase to the 3′-OH end of the invading strand [158]. When the D-loop reaction is conducted with human proteins, the RAD51AP1-UAF1 complex or BRCA1-BARD1 complex can be used in lieu of RAD54. These accessory factors of human RAD51 do not require ATP and act by facilitating the capture of the dsDNA partner to assemble a three-stranded nucleoprotein intermediate called the synaptic complex [135, 146, 147].

The use of negatively supercoiled dsDNA in the D-loop reaction helps ensure a high product yield (**Figure 9A** (3)) [157]. For this reason, it is imperative to examine the supercoiling state of the dsDNA substrate by native gel electrophoresis beforehand [159]. Moreover, all the protein preparations must be tested thoroughly for nuclease contamination that could digest either the ssDNA oligonucleotide or the supercoiled plasmid DNA [114, 159].

#### Repair DNA synthesis from a D-loop and its regulation

Extensive genetic evidence has implicated DNA polymerase (Pol) δ in repair DNA synthesis during DSBR by HR [160–163]. Reconstituted systems have been developed to examine the mechanism of the Polδ-mediated repair synthesis reaction [114, 164–167] (**Figure 9A** (4)). The reaction necessitates RFC-mediated loading of the polymerase processivity factor PCNA onto the primer-template junction in the D-loop structure. Polδ can synthesize up to several hundred bases of new DNA before being impeded by the topological stress that accumulates in the DNA template [114, 164]. In yeast cells, extensive repair DNA synthesis is dependent on the DNA helicase Pif1 [113, 114]. Accordingly, the addition of purified Pif1 (**Figure 9A** (5)), but not a helicase defective mutant of Pif1, stimulates not only the length of the DNA synthesis tract, but also the efficiency of utilization of the PCNA-bound primer end (**Figure 9B**) [114]. The stimulatory effect of Pif1 on repair DNA synthesis is contingent upon its physical interaction with PCNA [114, 168]. Interestingly, the extent of DNA synthesis is restricted by the DNA helicases Mph1 [116, 167]} and Srs2 [115]. It should be noted that Srs2 has a second function in DNA synthesis restriction via the disruption of SUMOylated PCNA-Polδ interaction [169].

#### Methods for the analysis of repair DNA synthesis products

The simplest analytical procedure involves the resolution of D-loops in which the ^32^P-labeled invading ssDNA strand has been extended by Pol δ by electrophoresis in a non-denaturing gel, followed by phosphorimaging analysis of the dried gel; the extended D-loops exhibit a slower mobility than the unmodified D-loop (**Figure 9B** (i)). Native gel analysis provides the means to monitor the dissociation of the extended invading DNA strand by a DNA helicase as well [114, 167, 170]. Routinely, a portion of the reaction mixtures is subject to gel electrophoresis under denaturing conditions. In this case, only the extended invading strand is detected upon phosphorimaging analysis (**Figure 9B** (ii)). For the quantitative measurement of DNA synthesis, the reaction is performed with D-loops made with an unlabeled invading DNA strand in the presence of a ^32^P-labeled deoxy-nucleotide during the synthesis phase. The reaction products are then resolved in a native or denaturing gel, followed by quantification of the labeled DNA species in the phosphorimager [114, 166].

The extent of repair DNA synthesis by Pol δ-PCNA-RFC is limited by the topological stress that accumulates in the extended D-loop structure. Accordingly, the addition of a topoisomerase to relieve the topological constraint allows a longer DNA synthesis tract to be made [164]. It has been demonstrated that extensive DNA synthesis seen in the presence of Pif1 occurs within the context of a migrating DNA bubble and is not affected by topoisomerase addition ([114]; **Figure 9B**). Electron microscopy coupled with metal shadowing can be used to reveal products of the “migrating bubble” mode of repair DNA synthesis [114].

### Assaying DNA Helicases for Processing Homologous Recombination Intermediates

**Figure 10:**
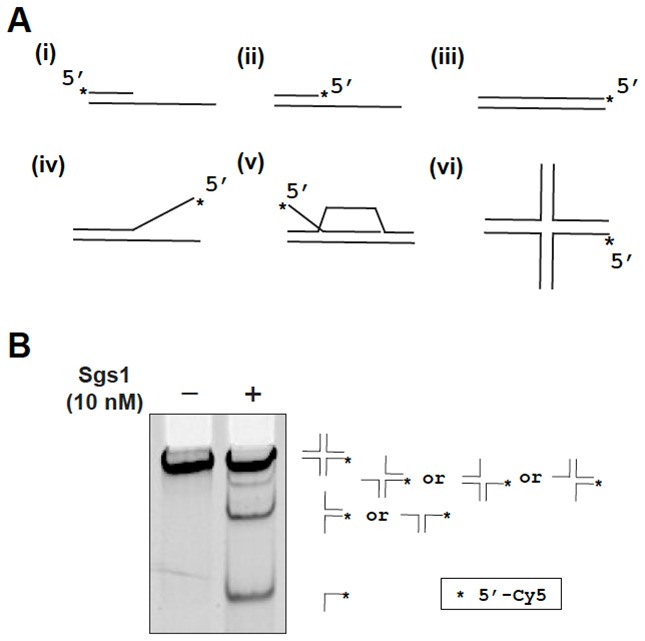
FIGURE 10: Holliday junction unwinding. **(A)** Diagrams of synthetic DNA structures frequently used in DNA helicase assays. **(B)** Sgs1-catalyzed unwinding of a fluorescently labeled Holliday junction substrate.

DNA helicase plays a pivotal role in the completion of HR-mediated DSBR. In vegetatively growing budding yeast, *S. cerevisiae*, as an example, multiple DNA helicases, including Sgs1, Srs2, Mph1 and Pif1, either directly participate in key steps of HR or process recombination intermediates for the choice among different HR pathways [109, 110, 113, 115–117, 123, 124]. The DNA helicases fulfill their functions largely through their capabilities of translocation on ssDNA or dsDNA in an ATP-dependent manner. The translocation of DNA helicases on ssDNA is associated with unique polarity (5′-3′ or 3′-5′), which allows a helicase to separate a duplex DNA into single strands. Due to their particular role in HR, a helicase frequently has its preferred substrate to unwind, e.g. a Holliday junction structure for the Sgs1 helicase [171]. Both the polarity and substrate specificity of a helicase can be examined *in vitro* by testing unwinding of various synthetic DNA substrates (**Figure 10A**). The assay system, once established, can also be applied to monitor the regulation of helicase activities by either cofactors or post-translational modifications.

#### Assembling substrates for DNA helicase assays

**1) Purification of oligos used for substrate assembling.** DNA substrates of various structures can be assembled with synthesized DNA oligonucleotides that are commercially available. In general, purification of synthesized oligonucleotides before substrate assembling by denaturing polyacrylamide gel electrophoresis (PAGE) is highly recommended, especially for oligos longer than 30 bases. Typically, a starting amount of 100–200 ug of oligo was used per purification. The oligos were first fractionated on a 20cm x 16cm format denaturing polyacrylamide gel containing 7M urea in TAE (40 mM Tris–acetate, 0.5 mM EDTA, pH 7.4) buffer with a BioRad Protean II system. Following electrophoresis, the bands of corresponding oligonucleotides can often be detected under long wavelength UV light without staining due to the UV shadowing effect. The bands are then excised and further sliced into small cubes, which are soaked in TE buffer overnight at 4°C. The extracted oligonucleotides are filter dialyzed and concentrated with Amicon Ultra Centrifugal Filters (EMD Millipore) before storage at – 20°C for future use.

**2) Labeling of oligonucleotides.** Generally, the helicase assays are conducted with substrate at low nanomolar concentration. For efficient detection and quantification, a selected strand, usually a product strand that is easily separable from the substrate through native PAGE, is labeled by either ^32^P isotope or a fluorescent dye. For structures comprised by multiple strands, e.g. a D-loop, substrates of the same structure but with different strands labeled individually can be constructed in parallel to examine the strand specificity of a particular helicase. The labeling can be at either 5′ or 3′ end of the target oligonucleotide. 5′ end labeling is applied more often and can be achieved by T4 polynucleotide kinase (PNK) catalyzed transfer of [γ-^32^P] phosphate from [γ-^32^P] ATP (PerkinElmer) to the 5′-OH end of target oligonucleotide. A synthesized oligonucleotide with a fluorescent dye conjugated at the 5′ end can be directly purchased from vendors at a cost. 3′ end labeling usually involves the addition of a radiolabeled nucleotide, e.g. [γ-^32^P] cordycepin 5′-triphosphate (PerkinElmer) or a fluorescence labeled nucleotide (Enzo Life Science) to the oligonucleotide via terminal transferase (New England Biolabs) catalyzed reaction. After labeling, a micro bio-spin 6 column (BioRad) is applied to remove the excess free nucleotides before the oligonucleotide is ready for hybridization reaction.

**3) Substrate assembling by hybridization.** The substrates for helicase assays are constructed by hybridizing the radiolabeled oligonucleotide to equimolar amounts of respective unlabeled oligonucleotide(s). The hybridization is achieved by slow cooling of oligonucleotide mixture in buffer 3 from New England Biolabs (50 mM Tris–HCl, pH 7.5, 10 mM MgCl_2_, 100 mM NaCl) from 95°C to room temperature. A preincubation at 95°C for 5 min is recommended to fully denature the oligonucleotides before hybridization. The whole procedure can be simply completed with a dry-bath heat block or a beaker of boiling water. The hybridized DNA substrate can be further purified from the excess un-annealed DNA by native PAGE. Notably, the electrophoresis should be conducted at 4°C to ensure the integrity of the substrate. The substrate DNA is extracted from the polyacrylamide gel similarly as described for oligonucleotide purification. Electroeulution of 1-2 hours at 4°C can be applied to extract substrates that are less stable, e.g. a D-loop structure.

#### Determination of helicase polarity by assays with overhanging DNA substrates

The DNA helicase unzips duplex DNA by translocation on ssDNA with a unique polarity. A helicase with 3′-5′ polarity preferentially unwinds the duplex DNA with a 3′ ssDNA overhang over the duplex DNA with a 5′ ssDNA overhang due to its 3′ to 5′ moving directionality on the ssDNA. A helicase with 5′-3′ polarity, on the other hand, favors duplex DNA with a 5′ ssDNA overhanging. Thus, helicase assays with duplex DNA bearing different overhangs can be applied to determine the polarity of a given helicase. As a start, helicase assays are typically setup with a 10 µl reaction system containing 20 mM Tris-HCl, pH 7.4, 2 mM ATP, 2 mM MgCl_2_, 50 mM KCl, 1 mM DTT, 100 µg/ml bovine serum albumin, substrate DNA at low nanomolar concentration and various amounts of the helicase enzyme (usually 1-100 nM). In the reaction, the concentration of radiolabeled substrate can be as low as 1 nM. The substrate labeled with fluorescent dyes has a lower sensitivity compared to radiolabeled substrate, thus a concentrate of 5 nM or higher is recommended. Helicase activities are usually sensitive to the concentrations of Mg^2+^ and KCl, therefore, titrations of both will help to determine the optimal condition for the given helicase. After the reaction (5-30 min at 30°C or 37°C), SDS (0.2%) and proteinase K (0.5 mg/ml) are added, followed by a 2 min incubation at 37°C, to stop and deproteinize the reaction. It should be noted that the unwound products may anneal spontaneously and regenerate the substrate DNA. Excess unlabeled form of the labeled oligonucleotide can be added following treatment with SDS and proteinase K to quench and stop the reaction. Unwinding of the substrate DNA can be examined by native PAGE analysis at 4°C. For assays with radiolabeled substrates, the gel is dried onto a sheet of Whatman DE81 paper (GE healthcare) followed by phosphorimaging analysis. For assays with fluorescent-dye labeled substrates, the gel can be scanned directly with the imaging system capable of fluorescence detection, e.g. an Amersham Typhoon 5 Imaging System (GE Healthcare). In addition to the aforementioned general procedures, there are two special notes on analyzing the fluorescent-dye labeled substrates by PAGE. 1) Before making the gels for the fluorescently labeled samples, the glass plates should be cleaned thoroughly with distill water and 70% ethanol for the minimal background signals. Following electrophoresis, the outer surfaces of the gel plates should also be cleaned before scanning. 2) Orange G is a preferred loading dye due to its minimal interference with fluorescent labeled substrates.

#### Helicase assays with more complex substrates

The procedures of helicase assays with overhanging DNA substrates depicted above can be readily applied to study substrates that are more complex. However, a certain DNA substrate with multiple single strands may be processed by a helicase in a step-wised manner to generate a series of intermediates, e.g. a Holliday junction structure unwound by Sgs1 (**Figure 10A, B**) [171]. Thus, enzyme titration and time course analysis are highly recommended to reveal the sequential order of product formation. For the substrate with an asymmetric nature, e.g. a D-loop substrate (**Figure 10A**), the respective single strands can be labeled individually to reveal the full product spectrum. Structure specific DNA unwinding by a helicase, sometime, may be regulated by a co-factor or a post-translational modification. Examination of these regulatory effects may require assay conditions that are more physiological, but less optimal. Titrations of parameters, such concentrations of KCl and Mg^2+^, can usually provide a guideline for setting up the assays.

To summarize, we have described a general procedure of helicase assays with DNA substrates that are either radiolabeled or labeled with a fluorescent dye. The radiolabeled substrate offers the best sensitivity, yet has essentially no modifications on the nucleotide structure, both of which are not offered by labeling with fluorescent dyes to the same level. Despite of these limitations, fluorescent labels have a few advantages as noted below. 1) DNA substrates labeled with fluorescent dyes has a long shelf-life when stored at -20°C in dark, which is extremely helpful for the initial substrate specificity test and for experiments with substrates that are relatively difficult to make, e.g. a synthetic double Holliday junction structure [118]; 2) The availability of fluorescent dyes with well-separated emission spectra enables multicolor labeling experiments, where multiple strands within a structure can be simultaneously labeled to probe the product spectrum; 3) The multicolor labeling also provides an opportunity to further develop the assay system for kinetic study based on FRET analysis.

### Fluorescence-based assays for structure-selective endonucleases

Structure-selective endonucleases make incisions on DNA strands of DNA joint molecules containing double-stranded and/or single-stranded DNA. Such DNA structures normally arise as intermediates of the major DNA metabolism pathways, such as DNA replication, DNA repair, single-strand annealing, and homologous recombination. In eukaryotes, most intensively studied are the structure-selective endonucleases Mus81-Mms4 (human MUS81-EME1/2), Slx1-Slx4 (human SLX1-SLX4/BTBD12), Rad1-Rad10 (human XPF-ERCC1), Rad2 (human XPG), Yen1 (human GEN1), and Rad27 (human FEN1), but additional enzymes exist and others may still be identified. For extensive review of structure-selective endonucleases see [172–176]. The *in vitro* analysis of endonucleolytic products produced by these enzymes allows the definition of their kinetic parameters, such as the values for *K*_M_ (the substrate concentration for at which the reaction rate is half of maximum velocity) and *k*_*cat*_ (the turnover rate of enzyme-substrate to enzyme-product) across a diverse set of DNA substrates as well as their sequence specificity or preference. These kinetic parameters provide quantitative insights into the substrate preference of individual enzymes [177]. Therefore, the information from such *in vitro* biochemical assays is fundamental to clarify the function of structure-selective endonucleases in DNA metabolism in living cells. Moreover, such *in vitro* assays can be employed to discover inhibitors that may serve to treat human disease [178, 179]. Here, we describe two methods to analyze *in vitro* the cleavage of fluorescent-labelled DNA joint molecules by structure-selective endonucleases. We previously described assays for structure-selective nucleases with radioactive-labelled oligo substrates [180]. The assays described here are based on FRET between two fluorophores [181] and are advantageous for laboratories who do not use radioactivity or have a need for a high-throughput format. The analysis can be performed in two different formats: through visualization of fluorescent-labelled DNA molecules by native acrylamide gel, analogous to radio-labelled substrates, or through the inhibition of FRET between two compatible fluorophores attached to opposite sides of the cleavage position on the DNA substrate.

#### Description of Assay

Different kinds of joint DNA molecules are made by the annealing of partially complementary oligonucleotides of which two are fluorescent-labeled. We describe in **Table 1** different substrates and their respective constituent oligonucleotides and sequences that are recognized and cleaved by structure-selective endonucleases. For oligonucleotide annealing conditions and substrate production, we refer the reader to [182]. For instance, the oligos olWDH953 (5′- TCTGACTGCAGTCGGGCT-3′), olWDH1388 (5′ACCGTCCGTCCTAGCAAGCATTCGAT/3Cy5sp/-3′), and olWDH1390 (5′AGCCCGACTGCAGTCAGAGCTTGCTAGGAC GGA/iCy3/CGGT-3′) compose the substrate Cy3/Cy5 labeled 3′-flap (called Cy3/Cy5 3′-flap) [183]. The Cy3/Cy5 3′-flap substrate can be visualized as the higher band after the annealing step in a native 10% acrylamide gel. Because it contains both Cy3 and Cy5 labelling, the gel band can be visualized either by the Cy3 or Cy5 filters, or by the merged image captured by both filters.

**Table 1.**
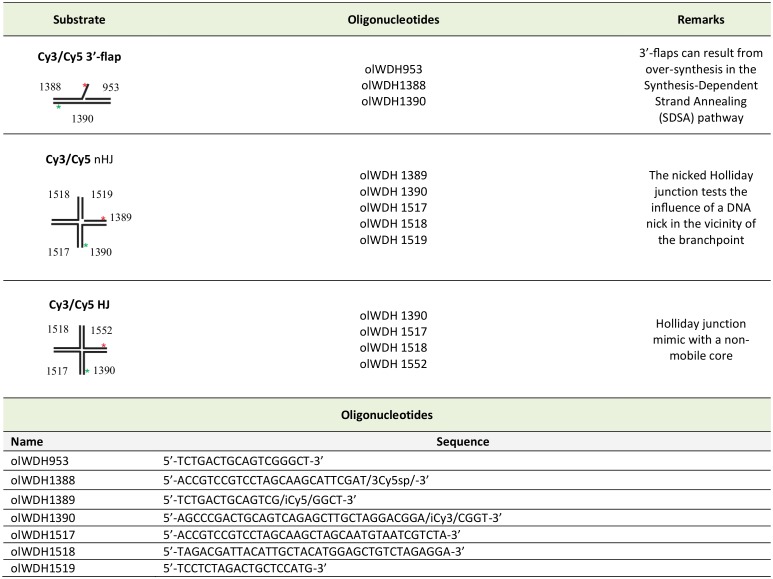
Substrates and their oligonucleotide names and sequences. Cy3 and Cy5 fluorophores are usually at the 3′ end.

**Figure 11:**
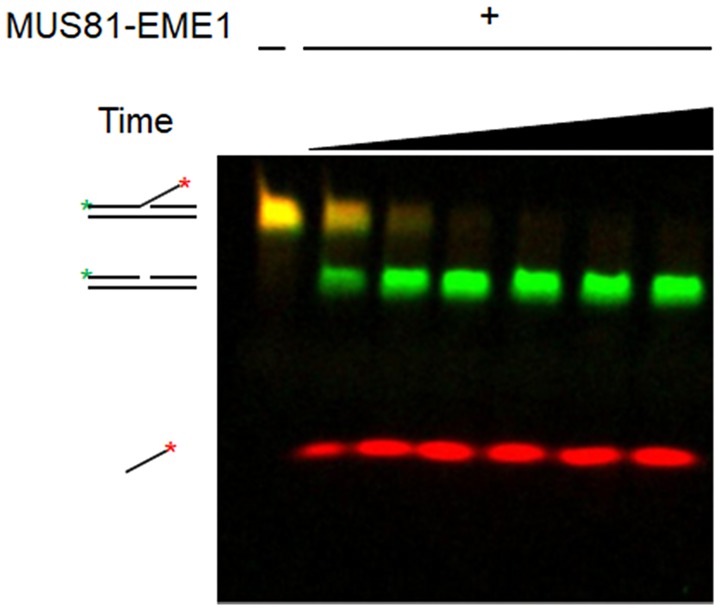
FIGURE 11: Gel-based endonuclease assay using a fluorescent substrate. Time course analysis for 1, 3, 5, 7, 10, and 15 minutes with 100 nM of the Cy3/Cy5 3′-flap substrate and 5 nM human MUS81-EME1 visualized on a 10% native acrylamide gel with Cy3 and Cy5 filters.

**Figure 12:**
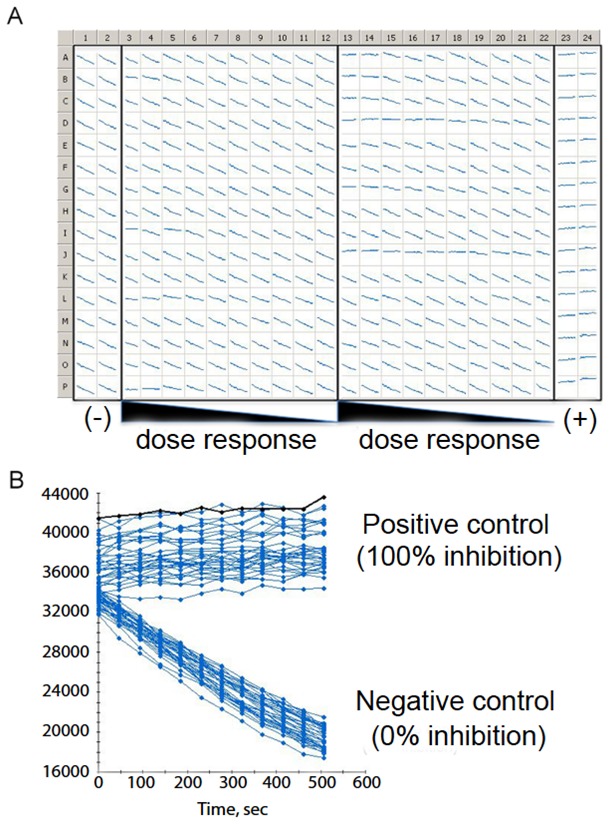
FIGURE 12: FRET-based endonuclease assay. **(A)** A representative 384-well plate view kinetic measurement of the FRET-based MUS81-EME1 assay, with compounds tested in 10-concentrations dose response. **(B)** Graph shows the slope / reaction rate for assay controls. Kinetic readout (Excitation 535 nm / Emission 665 nm) for 8 minutes at linear phase was measured per assay plate and data was calculated as slope from a time series of 12 data points representing the rate of reaction. The assay was robust with Z' performance of 0.74 ± 0.04.

The cleavage of the joint DNA molecules can also be visualized on a 10% native acrylamide gel (**Figure 11**). For example, human MUS81-EME1 cleaves the ssDNA of the Cy3/Cy5 3′-flap substrate. The original substrate can be visualized by a native 10% acrylamide gel with filters for Cy3 and Cy5 as the higher yellow band. The products of endonuclease cleavage can be visualized as the smaller green (Cy3-labelled nicked dsDNA) and red bands (Cy5-labelled ssDNA) (**Figure 11**). Optimized reaction conditions for human MUS81-EME1 are 25 mM Tris, pH 8, 50 mM KCl, 5 mM Mg(OAc)_2_,1 mM DTT, 0.1 mg/mL BSA, 100 nM of substrate, 5 nM of enzyme, at 37°C. The endonuclease reaction can be also detected in real-time by the FRET-based assay. In the 3-flap molecule for example, the Cy3 group will absorb and emit fluorescence at 535 and 572 nm, respectively. Because of the proximity between Cy3 and Cy5 groups, the energy emitted from Cy3 is absorbed by Cy5 that will then emit fluorescence at 665 nm. Therefore, fluorescence measurements performed at λ_ex_ =535 nm and λ_em_ = 665 nm can either detect FRET between the pair Cy3/Cy5 or the FRET inhibition caused by the endonuclease cleavage (**Figure 12**). Reactions of FRET-based assays are compatible with low volume 96/384-well black microplates, flat-bottom. The conditions are similar to those described for the gel-based assay: 2.5 nM protein, 25 nM Cy3/Cy5 3′-flap, 8 min at room temperature, 25 mM Tris-HCl, 50 mM KCl, 5 mM Mg(CH3COO)_2_, 0.1 mg/mL BSA, 0.01% Triton, 10 mM BME. The FRET-based assay is suitable for high throughput screenings for the discovery and validation of small molecule inhibitors for structure-selective endonucleases. Dose response curves can also be performed to determine the IC_50_ values of different inhibitors, usually within concentrations ranging from 0.1 to 100 µM.

Both assays, the gel-based and the FRET-based, can be designed as a single end point or through a time course with several time points. End point assays can be used to determine minimal substrates or optimal cleavage conditions as a function of different salt, buffer type or pH. Through time course analysis it is possible to determine kinetic parameters as *K*_M_ and *k*_cat_. For instance, for the Cy3/Cy5 3′-flap and nicked Holliday junction (nHJ) substrates with human MUS81-EME1, initial reaction velocities are calculated from progression curves from 0, 1, 3, 5 min time points with 10, 20, 50, 100, 150, 200 nM substrates and 5 nM heterodimer. The derived *K*_M_ values with human MUS81-EME1 are 30 ± 3.5 nM for the Cy3/Cy5 3′-flap substrate and 11 ± 4.9 nM for the Cy3/Cy5 nHJ substrate, indicating a roughly 3-fold higher affinity of MUS81-EME1 for the nHJ over the 3′-flap substrate, consistent with published data using radio-labelled substrates [184].

#### Cautionary notes

Proper quality control should be performed to test the purity of the enzymes used in the above assays. It is critical to track the presence of contaminating DNA, as the presence of DNA in the enzyme preparation affects the behavior towards test substrates. Standard purity tests of enzyme preparations include staining of purified fractions resolved by gel electrophoresis or analysis by mass spectrometry. In addition, functional assays to detect the presence of contaminating activities is important, as such enzymes act catalytically and minute contamination can interfere with assay. Depending on the specific enzyme purified such potentially interfering activities include endonucleases (in case of structure-selective endonuclease circular ssDNA and dsDNA), exonucleases, phosphatases, topoisomerases (when circular substrates are used), or polymerases. The assays for quality control of purified enzymes involved homologous recombination has been described in [185]. An excellent control is the catalytic inactive version of the nuclease of interest, in particular when more complex reactions are reconstituted that contain more than one protein.

Activity assays need to be optimized for concentration and type of the metal ion, pH, concentration and type of salt as well as type of buffer, and potentially the presence of crowding agents. The optimization of the reaction conditions should be performed before the kinetics analysis to assure the enzyme will reach the maximum initial velocity during determination of the kinetic parameters. Definition of the optimal reaction conditions is particularly pertinent in reconstituted reactions with multiple enzymes, which may differ in their optima.

The concentration of enzyme and substrate should be converted from mass/volume to molar units. Reactions should be set up with excess substrate relative to the enzyme concentration and typical stoichiometries for reactions involving structure-selective endonucleases are 1 endonuclease to 5-20 joint DNA molecule substrates. From this discussion, it is evident that immunoprecipitates as a source of an enzyme does not allow quantitative comparison of substrate preference because the enzyme concentration is unknown resulting likely in vast excess of enzyme over substrate, when pure radiolabeled substrate is being used.

End point assays are informative to determine minimal substrates or optimal reaction conditions. Indeed, if reactions are visualized on denaturing gel, endpoint reactions can be used to define the site of incisions catalyzed by structure-selective endonucleases [180]. However, end point assays are less informative to define kinetic parameters because initial maximum velocity is more accurately measured over a time course.

During high throughput screening (HTS), controls are typically embedded with sufficient replicates within each of the microplates tested to evaluate assay performance. Negative controls (0% inhibition), representing full enzymatic reaction, are wells consist of all reaction components. Positive controls (100% inhibition) can be the inhibitory reaction by a known inhibitor. Mock positive controls, where enzyme is excluded in the reaction mixture, can be employed to mimic a full inhibition when there is a lack of suitable inhibitor. The FRET-based assay by kinetic readout should complete within 5-10 minutes per 384-well plate (*i.e.* 6-12 plates per hour). The time is often a compromise between achieving good throughput during an HTS campaign and capturing sufficient data-time points to enable statistically reliable calculation for the rate of the reaction. Screening actives are typically selected from test data showing inhibitory effect with 2-3 standard deviation away from the mean value of negative controls (0% inhibition). These actives are then tested in replicative dose response of multiple concentrations. Confirmed hits that warrant further investigations are compounds showing potent sub-micro molar IC_50_ with near 1:1 enzyme-inhibitor stoichiometry. Although FRET measurement, based on changed emission of donor and acceptor, is typically ratiometric, it can still be skewed by excessive autofluorescence from some test compounds. It is therefore a good practice to measure the pre-reaction fluorescence of each wells treated with compounds. Screening hits from wells with compounds with excessive high fluorescence should be examined as potential false positives.

#### Conclusion

Structure-selective endonucleases are an important class of enzymes in all aspects of DNA metabolism. Determining their inherent substrate preference and cleavage patterns provides important information to establish the *in vivo* function of these enzymes. Here, we describe two assay formats using fluorescently-labeled DNA substrates paired with gel electrophoresis or FRET analysis that allow kinetic and endpoint analysis of structure-selective endonuclease independent of using radioactivity and compatible with high-throughput screening.

## SUMMARY

The maintenance of genomic information and genomic integrity lies at the core of all organismal propagation, development and survival. The past two decades have witnessed the emergence of several new and powerful physical approaches collectively termed single-molecule techniques. Utilizing these methods for studying biological systems provides many new features that are otherwise masked due to averaging in ensemble measurements, thus providing previously unattainable data and new mechanistic insights. Herein we provide a concise description and procedures on the use of several next-generation single-molecule techniques, assays and tools that are used to study key molecular mechanisms and pathways in DNA repair, and address fundamental questions in the field. These include methods such as super-resolution localization microscopy for real-time tracking of individual molecules in live cells, single-molecule tracking in vitro assays, single-molecule manipulation, and single-molecule FRET. Beyond their practical description, we sought to highlight both the strengths and limitations of each technique to give in context explanations of how each method should be employed to investigate DNA repair mechanisms.

## SUPPLEMENTAL MATERIAL

Click here for supplemental data file.

All supplemental data for this article are available online at http://www.microbialcell.com/researcharticles/2019b-klein-microbial-cell/.

## Abbreviations:

2-AP: 2-aminopurine,
BER: base excision repair,
CMOS: complementary metal-oxide-semiconductor,
dHJ: double Holliday junction,
D-loop: displacement loop,
DSB: double-strand break,
DSBR: double strand break repair,
dsDNA: double stranded DNA,
EMCCD: electron charge coupled device,
FP: fluorescent protein,
FRAP: fluorescence redistribution after photobleaching,
FRET: Förster resonance energy transfer,
FROS: fluorescent repressor operating system,
GFP: green fluorescent protein,
HR: homologous recombination,
HMM: hidden Markov model,
HTS: high throughput screening,
MMR: mismatch repair,
MMS: methyl methanesulfonate,
MSD: mean-square displacement,
NER: nucleotide excision repair,
NHEJ: nonhomologous end joining,
OD: optical density,
PAFPs: photoactivable fluorescent proteins,
PALM: photoactivated localization microscopy,
QDots: quantum dots,
RNAP: RNA polymerase,
SDSA: synthesis-dependent strand annealing,
smFRET: single molecule Förster resonance energy transfer,
ssDNA: single stranded DNA,
SPT: singleparticle tracking,
TCR: transcription coupled repair,
TIRF: total internal reflection fluorescence,
TLS: translesion synthesis,
UV: ultraviolet,
WT: wild type,
YFP: yellow fluorescent protein.
