# Supplementary material for: Guidelines for DNA recombination and repair studies: Mechanistic assays of DNA repair processes
Source: Microb Cell. 2019 Jan 7;6(1):65–101. doi: 10.15698/mic2019.01.665 (PMC6334232; doi:10.15698/mic2019.01.665)
Supplement: Supplementary file 1 [file mic-06-065-s01.pdf]

Title	Authors	Contact information of lead authors
1. Dancing on DNA tightropes: watching repair proteins interrogate DNA in real time.	Emily C. Beckwitt, Bennett Van Houten	vanhoutenb@upmc.edu
2. Single-molecule FRET illuminates the non-homologous end joining process in vitro	Michael J. Morten, Eli Rothenberg	eli.rothenberg@nyumc.org
3. Single-molecule imaging measures dynamics and localization to uncover the mechanism of DNA mismatch repair in living cells	Julie S. Biteen	jsbiteen@umich.edu
4. Single-molecule DNA nanomanipulation	Shuang Wang, Terence R. Strick	strick@ijm.univ-paris-diderot.fr
5. Single-molecule PALM imaging of translesion polymerases in live bacterial cells	Elizabeth S. Thrall, Joseph J. Loparo	joseph_loparo@hms.harvard.edu
6. Tracking-PALM: a direct single-molecule imaging method to study DNA repair in living bacteria	Oliver Pambos, Jun Fan, Hafez el Sayyed, Achillefs Kapanidis	achillefs.kapanidis@physics.ox.ac.uk
7. Biochemical analyses of recombinase filament assembly	Hsin-Yi Yeh, Yi-Hsuan Chang[1], and Peter Chi	peterhchi@ntu.edu.tw
8. Assaying DNA repair synthesis by DNA polymerase δ and its stimulation by Pif1 helicase	Youngho Kwon, Patrick Sung	Patrick.sung@yale.edu
9. Assaying DNA Helicases for Processing Homologous Recombination Intermediates	Quan Wang, Hengyao Niu	hniu@indiana.edu
10. Fluorescence-based assays for structure-selective endonucleases	João P. Vieira-da-Rocha, Kenny Ang, Sucheta Mukherjee, Michelle R. Arkin, Wolf-Dietrich Heyer	wdheyer@ucdavis.edu
